# Brain information processing capacity modeling

**DOI:** 10.1038/s41598-022-05870-z

**Published:** 2022-02-09

**Authors:** Tongtong Li, Yu Zheng, Zhe Wang, David C. Zhu, Jian Ren, Taosheng Liu, Karl Friston

**Affiliations:** 1 Department of Electrical and Computer Engineering, Michigan State University, East Lansing, MI 48824 USA; 2 Department of Radiology, Michigan State University, East Lansing, MI 48824 USA; 3 Department of Psychology, Michigan State University, East Lansing, MI 48824 USA; 4 The Wellcome Centre for Human Neuroimaging, University College London, London, UK

**Keywords:** Neuroscience, Engineering

## Abstract

Neurophysiological measurements suggest that human information processing is evinced by neuronal activity. However, the quantitative relationship between the activity of a brain region and its information processing capacity remains unclear. We introduce and validate a mathematical model of the information processing capacity of a brain region in terms of neuronal activity, input storage capacity, and the arrival rate of afferent information. We applied the model to fMRI data obtained from a flanker paradigm in young and old subjects. Our analysis showed that—for a given cognitive task and subject—higher information processing capacity leads to lower neuronal activity and faster responses. Crucially, processing capacity—as estimated from fMRI data—predicted task and age-related differences in reaction times, speaking to the model’s predictive validity. This model offers a framework for modelling of brain dynamics in terms of information processing capacity, and may be exploited for studies of predictive coding and Bayes-optimal decision-making.

Characterizing the information processing capacity of the human brain is a key challenge in cognitive psychology and neuroscience. Most of the existing research in this area has focused on the capacity limit of short-term working memory, or how well an individual handles information processing demands when several tasks have to be executed simultaneously^1–5^. It is thought that our visual short-term memory can maintain representations of three to four objects at any given moment^6^. Along this line, in^7^, information processing capacity was mapped to the computational capacity of a dynamic system and characterized as the total number of linearly independent functions of input stimuli the system can compute.

Previous research in neurophysiology suggests that human information processing is reflected in neuronal activity^8–10^. For example, the amplitude of neuronal activity is modulated by the input information flow or the number of objects held in memory, before approaching an asymptotic limit^8,9^. Furthermore, automation of cognitive functions can increase capacity and attenuate neuronal responses^10^.

Early quantitative characterizations of neuronal activity considered the computational properties of the spikes of single neurons. The first formal neuron model was the Hodgkin–Huxley model, which was based on detailed neurophysiological recordings of the squid giant axon^11^. While this model can reproduce electrophysiological measurements accurately, it is intrinsically complex and difficult to analyze. To reduce model complexity, simple statistical spiking neuron models—represented by the integrate-and-fire (IF) models—were developed, by replacing the rich dynamics of the Hodgkin–Huxley formulation with a simple fire-and-reset process^12^. Refined with the time elapsed since the last spike, the ensuing generalized IF models were shown to have high accuracy in predicting the responses of real neurons^13^.

In light of the Hodgkin–Huxley models and IF models, dynamic models for collective activity were subsequently developed by exploiting the interactions of excitatory and inhibitory cells, and the coherence of neural populations^14–16^, or by approximating the cumulative activity of the neural population as a Gaussian random process based on the central limit theorem^17–19^. A comprehensive review of dynamic models for population or ensemble activity can be found in^20^.

Moving from the modeling of the collective activity of neuronal populations (or individual brain regions) to the characterization of network-level activity of connected brain regions, dynamic neural system models were developed^21,22^. A representative network-level model is used in dynamic causal modeling (DCM)^22–24^, which models neuronal dynamics in terms of the (intrinsic) self-connectivity within each region and the (extrinsic) cross-connectivity among regions based upon neural mass formulations. Recently, network models—based on graph theory and entropy—have also been used to characterize the directed information flow and integration in large-scale brain networks^25,26^. In summary, existing models of neuronal activity offer a panoramic coverage of brain dynamics, from the single neuron, through neural populations, to brain networks. However, under all these models, the quantitative relationship between the activity of a brain region and its information processing capacity remains unclear.

In this article, we considered neuronal activity and information processing capacity from an information-theoretic perspective. Starting from an information conservation law, we showed that for an individual brain region, the information processing capacity, input storage capacity, neuronal activity, and the arrival rate of exogenous information can all be related through a first-order differential equation. Theoretically, our model indicates that the difference between the information arrival rate and the information processing rate directly influences neuronal activity changes. Higher information arrival rate enhances neuronal activity, while larger processing capacity decreases neuronal activity; on the other hand, larger input information storage capacity can alleviate the demand on neuronal activity, when the arrival rate increases.

We applied this model to an empirical fMRI dataset, which was acquired under a rapid event-related arrow flanker task—used to study aging-associated decline in selective attention and executive functions. Both young and old adult groups participated in the experiment. We analyzed individual brain regions that were activated in both the young and old groups. We also considered overall information processing by averaging the data from each region. Our numerical analysis demonstrated the accuracy of the model in explaining fMRI measurements and showed that—for a given cognitive task—higher information processing capacity engenders a lower neuronal activity and faster response in younger subjects. That is, younger adults have faster responses and better performance in the flanker test than the seniors, because they have higher information processing capacity. This result is consistent with findings in literature suggesting that high-capacity individuals tend to have lower neuronal activity^8,9^, and that—compared with young adults—more brain activation was required for older adults to accomplish the same cognitive task^27,28^. Crucially, these findings speak to the predictive validity of the model, in the sense that we were able to predict the behavioral responses from (independent) fMRI responses.

While the information processing capacity (IPC) model is a novel formulation, it is reassuring that—although originating from information theory—our model has a similar functional form to the conductance-based neural mass models in DCM, as well as the IF model of individual neurons. The implication here is that—with an information conservation law as the cornerstone—our model is not limited to brain regions, but can be applied to any neuronal system that has the attributes of information processing and storage capability. In sum, the model offers a framework for multiscale modelling of brain dynamics in terms of information processing and provides a new perspective on computational architectures in the brain. And it can be applied to any data from which neuronal activity can be estimated.

*The notations used throughout this article are provided in Table 1.

**Table 1:** Notation table.

Notation	Meaning	Notation	Meaning
$y(t)$	BOLD Signal	$m/\alpha$	Relative input storage capacity
$x(t)$	Neuronal activity	$p$	Information processing capacity (in bit/sec)
$h(t)$	Hemodynamic response function (HRF)	$p/\alpha$	Relative information processing capacity
$\alpha_{1}$	Response delay of the HRF	$T_{y}$	Time to peak of the BOLD signal
$\alpha$	Input information (in bits)	$T_{c}$	Time constant of the (regional) brain circuit (in seconds)
$\alpha_{C}$	Input information under Congruent condition (in bits)	$T_{0}$	Onset time of the inhibitory neuronal activity (in seconds)
$\alpha_{IC}$	Input information under Incongruent condition (in bits)	$T_{1}$	Onset time of the secondary excitatory neuronal activity (in seconds)
$m$	Input storage capacity (in bits)	$T_{r}$	Average response time of the subject group observed in the experiment (in seconds)

## Results

### Information conservation in a lossless brain region

In a *functional* sense, we can claim that any neuronal system (e.g., brain region) has a processing unit and an input storage unit, which can vary across time and/or on-going cognitive tasks. Let *I*(*t*) denote the overall information delivered to a brain region during the period $\left[ {0, ~t} \right]$; $I_{p}(t)$ be the information taken by the processing unit to process during [0, *t*], and *I*_*m*_(*t*) the information saved in the input storage unit at time instant *t* that is waiting to be processed.

We start with the *lossless* situation, that is, there is no information loss in the region. In this case, we have: *the total information delivered to the region equals the sum of the information taken for processing, and the information saved in the input storage unit.* That is,

1 $$I(t) = I_{p} (t) + I_{m} (t),$$

all in *bits*. We refer to this as the ***information conservation law**** in a lossless region*. It should be emphasized that our input storage unit here holds only the input information waiting to be processed, and is just a functional construct used to simplify the analysis. The memory request that occurs during the processing operations is considered to be part of the processing rather than the storage. As demonstrated below, the information conservation law—a simple but fundamental principle—serves as the bridge between the information processing and storage capacities of a brain region and its neuronal activity level.

### Representing the neuronal activity in terms of information processing and storage capacities

Taking derivatives with respect to $t$ on both sides of Eq. (1), we get $H(t) = H_{p}(t) + H_{m}(t)$ where $H(t) = dI(t)/dt$ is the *information arrival rate*, $H_{p}(t) = dI_{p}(t)/dt$ is the *information processing rate* and $H_{m}(t) = dI_{m}(t)/dt$ is the *instantaneous information rate in the storage unit,* all in bits per second.

Let *p*(*t*) represent the processing capacity, defined as the maximal information processing rate (in bit/s) of the region with respect to a particular cognitive task. Let $m(t)$ represent the input storage capacity, defined as the total amount of input information storage resource (in *bits*) available to, or allocated by, the region.

Recall that the neuronal activity $x(t)$, which reflects the computational cost (or effort level) of fulfilling a cognitive task or function, can be understood as the instantaneous percentage of the total “workforce” being utilized in the neuronal population or region. From an information processing perspective, (normalized) neuronal activity can be defined as the ratio of the instantaneous processing rate and the maximum processing rate: *x*(*t*) = *H*_*p*_(*t*)/*p*(*t*). This reflects how actively the processing unit is ‘working’ to fulfill the cognitive task. Similarly, from the storage perspective, (normalized) neuronal activity can be defined as: *x*(*t*) = *I*_*m*_(*t*)/*m*(*t*), which is the ratio between the actual input information saved in the input storage unit and the overall storage capacity.

### A first-order differential equation model for neuronal activity

We consider a single state brain region, that is, a region whose activity is summarized with one neuronal activity level at any given time instant. We adopt a single-state model by appealing to Landauer’s principle and, specifically, the Jarzynski equality^29–31^, which suggests that there is a singular thermodynamic cost for the processing of information. For a brain region, this cost is generally thought to be reflected in the hemodynamic responses measured with fMRI^32–35^. Practically, we treat the average BOLD signal from voxels in the region as a measure of this cost $x(t),$ over the region in question. In other words, for a single-state brain region, input storage and processing are underwritten by the same neuronal activity level. Moreover, when the task itself has a sufficiently short duration, we can assume that the processing capacity *p* and the storage capacity *m* remain unchanged throughout the task (i.e., an adiabatic approximation). In this case, we have

2 $$\frac{dx(t)}{{dt}} = \frac{1}{m}\frac{{dI_{m} (t)}}{dt} = \frac{1}{m}\left[ {H(t) - H_{p} (t)} \right]$$

and $x(t) = \frac{{H_{p} (t)}}{p}$; it then follows that the neuronal activity of an individual brain region can be modeled as:

3 $$\frac{dx(t)}{{dt}} = - \frac{p}{m}x(t) + \frac{1}{m}H(t).$$

This furnishes a first-order linear differential equation that connects the neuronal activity of a brain region with its information processing, storage capacities and the information arrival rate. We refer to it as the ***Information Processing Capacity (IPC) model***. In the special case when $I(t) = \alpha u(t)$ is a step input, where $u(t)$ is the unit step function, we have $H(t) = \alpha \delta(t)$, where $\delta \left( t \right)$ is the Dirac delta function, and then $x(t) = \frac{\alpha }{m} e^{{ - \frac{t}{{T_{c} }}}} u(t)$, with $T_{c} = \frac{m}{p}.$ As can be seen, $x( {0_{ - } } ) = 0$ and $x(0) = \frac{\alpha }{m}$, that is, there is an abrupt change of the neuronal activity at $t = 0.$ This is consistent with the findings in literature that neuronal responses to new sensory information show a phasic or onset response. For example, neural engagement increases abruptly at the start of learning, and then gradually declines [36].

From Eq. (2), the interpretation of this first-order IPC model is as follows: the gap between the information arrival rate $H(t)$ and the information processing rate $H_{p}(t)$ directly determines the changing rate of the neuronal activity. When $H(t) > H_{p} (t)$, that is, when the arrival rate is higher than the processing rate, then the neuronal activity level will increase; otherwise, it will decrease. On the other hand, a greater input information storage capacity *m* can alleviate the demand on neuronal activity, when the arrival rate *H*(*t*) increases. This affords the potential for adaptation and self-adjustment that is a ubiquitous aspect of neuronal processing.

### An equivalent brain circuit model of excitatory and inhibitory interactions

**Figure 1:**
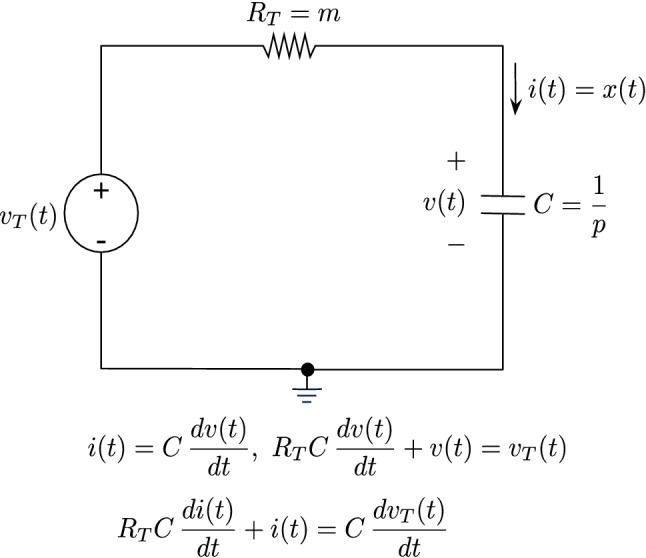
A resistor–capacitor (RC) circuit model for neuronal activity.

As is well-known, the first-order differential equation model above can be associated with a resistor–capacitor (RC) circuit as shown is Fig. 1, where $v_{T}$ and $R_{T}$ denote the Thévenin equivalent voltage and resistance, respectively, and $x(t)$ can be regarded as the current that goes through the RC circuit.

We say that $x(t)$ is the current—as opposed to the voltage—across the capacitor, since $x(t)$ experiences an abrupt change at $t = 0,$ and the voltage across the capacitor is a continuous variable and therefore cannot change abruptly. Compare Eq. (3) and the differential equation corresponding to the RC circuit, if $v_{T}(t) = I(t),$ then we have $R_{T} = m, C = \frac{1}{p}$, and the time constant of the circuit is $T_{c} = R_{T} C = \frac{m}{p}$, which is related to the response time of the brain region.

Recall that—based on experimental observations of neuronal networks—neurons constantly integrate excitatory activity and inhibitory input over both time and the dendrites receiving synaptic contacts^37–39^. In other words, postsynaptic responses reflect the superposition of the excitatory activity and inhibitory input^37,39^. Recent studies^24^ suggest that neural mass models equipped with both excitatory and inhibitory neuronal populations provide a better account of BOLD (fMRI) responses than models based upon a single (lumped) neuronal state. To accommodate a more expressive model of information processing—that integrates both inhibitory and excitatory neuronal activity—let $I_{E}(t) = I(t)$ denote the excitatory input information as before, and $I_{I}(t)$ the inhibitory control signal, then we have $v_{T}(t) = I_{E} (t) - I_{I}(t).$ We start with a simple case and model this superposition through the circuit in Fig. 1 as *a switched circuit* with $v_{T}(t) = \alpha u(t) - \beta u( {t - T_{0} } ).$

The interpretation here is that, under a stimulus, the excitatory input information lasts a period of $T_{0}$ seconds before it is subverted by an inhibitory control signal. The first transition in $v_{T}(t)$ occurs at $t = 0,$ and can be considered as the onset response to an input stimulus; the latter can be considered as clearing the previous input data and preparing for new input: a process driven by a negative feedback control mechanism to prevent the brain from excessive activation^39^. Based on the superposition property of linear circuits, the overall neuronal activity $x(t) = x_E(t) - x_I(t),$ where $x_E(t) = \frac{\alpha }{m} e^{{ - \frac{p}{m} t}} u\left( t \right)$ is the excitatory activity and $x_I(t) = \frac{\beta }{m} e^{{ - \frac{p}{m} \left( {t - T_{0} } \right)}} u\left( {t - T_{0} } \right)$ is the inhibitory activity.

Note that excitatory inputs and inhibitory control signals can occur multiple times^38^. Recent studies indicate that excitation–inhibition balance (E–I balance) is a form of homeostatic plasticity that helps to maintain neuronal activity within a safe physiological range^40,41^. We can incorporate this dynamic by extending the two-state model above to: $x(t) = x_E(t) - x_I(t),$ where $x_E(t) = \mathop \sum \limits_{i = 0}^{M} \frac{{\alpha_{i} }}{m} e^{{ - \frac{p}{m} \left( {t - T_{E,i} } \right)}} u\left( {t - T_{E,i} } \right)$ is the excitatory activity and $x_I(t) = \mathop \sum \limits_{j = 0}^{N} \frac{{\beta_{j} }}{m} e^{{ - \frac{p}{m} \left( {t - T_{I,j} } \right)}} u\left( {t - T_{I,j} } \right)$ is the inhibitory activity.

We now have a physiologically plausible model of evoked neuronal responses that are parameterized in terms of information processing capacity and storage capacity. This means that when estimating the shape of evoked responses from empirical data, the ensuing parameter estimates have a direct interpretation in terms of information processing. In what follows, we apply this formalism to event related responses, in which we try to recover the neuronal responses from fMRI timeseries.

### Model inversion and validation using fMRI data

We applied the IPC model in Eq. (3) to characterize neuronal activity in terms of information processing capacity using fMRI data obtained from a flanker test, which was used to study the aging-associated decline in selective attention and executive function^42^. Twenty-three young adults and twenty-six older adults participated in this study. In the experiment, the subjects were presented with three conditions: the Congruent (C) condition (“>>>>>>>” or “<<<<<<<”), the Incongruent (IC) condition (“>>><>>>” or “<<<><<<”) and the Neutral condition (“□□□ > □□□” or “□□□ < □□□”). Each trial was presented for 2.5 s, during which time the subjects were asked to identify the direction of the central arrowhead and press the corresponding button for each trial. The rapid event-related design was chosen so that the subjects’ general attentiveness level was kept relatively constant. More details can be found in the Method section. For model verification, we analyzed *the middle frontal gyri* (MFG), *the inferior frontal gyrus* (IFG), *the inferior occipital gyrus* (IOG), *the middle occipital gyrus* (MOG), and *the superior frontal* *gyrus* (SFG). All the regions were inferred to be activated using standard fMRI data analysis procedures.

We considered both fixed and flexible hemodynamic response functions (HRFs) when estimating information processing parameters, i.e., we used the same (fixed) HRF for all groups or a separate HRF for each group, using standard maximum likelihood estimates. The basic idea was to identify the $h(t)$ that minimized the mean-square-error (MSE) between the true BOLD signal $y(t)$ and the estimated BOLD signal $y_{est}(t) = x_{est}(t) *h(t),$ where $x_{est}(t)$ was obtained for the selected $h(t)$ using the Least-Square method. For fixed HRF, $h(t)$ was estimated with respect to the old group (under the Incongruent condition) and was assumed to be invariant over each region, young and old groups, and Incongruent and Congruent Conditions. For Flexible HRFs, $h(t)$ was estimated for each group (Young vs. Old) and condition (Congruent vs. Incongruent).

We found that the response delay of the HRF has a greater impact on the MSE than the undershoot delay, since the peak response has a larger amplitude than the undershoot. However, provided the peak delay is within an appropriate range, small MSEs (generally $\sim 10^{ - 4}$ or smaller) were obtained. This explains why we obtained similar information processing results, under fixed and flexible HRFs. Please refer to the Methods section and the supplementary material for more details.

**Figure 2:**
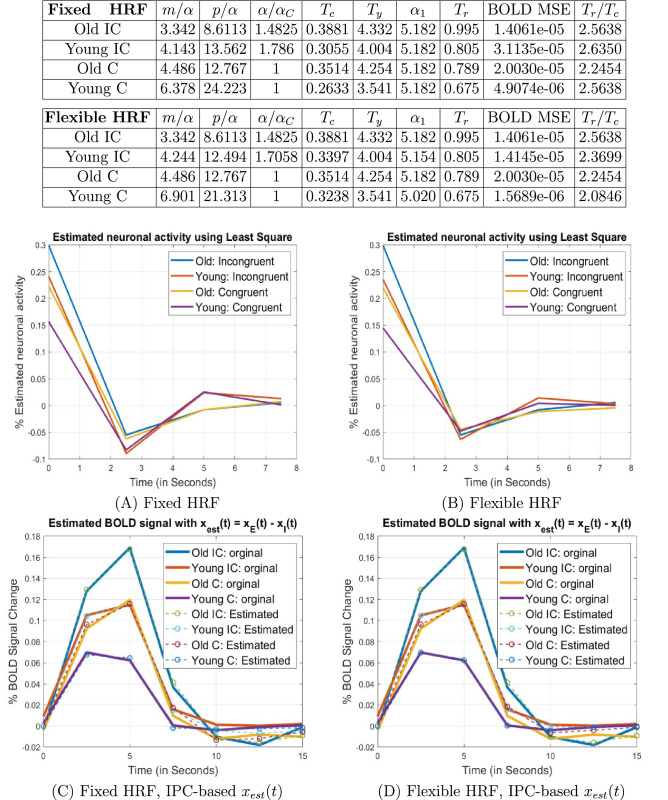
RMFG young: Analysis results for the right middle frontal gyrus (RMFG) region found in the young group. The parameter estimation results indicated that compared with the old group, the young group has higher relative information processing capacity under the same task. In (C) and (D), the estimated BOLD signal $y_{est}(t) = x_{est}(t)*h(t),$ where $x_{est}(t) = x_E(t) - x_I(t),$ was obtained based on the IPC model.

**Figure 3:**
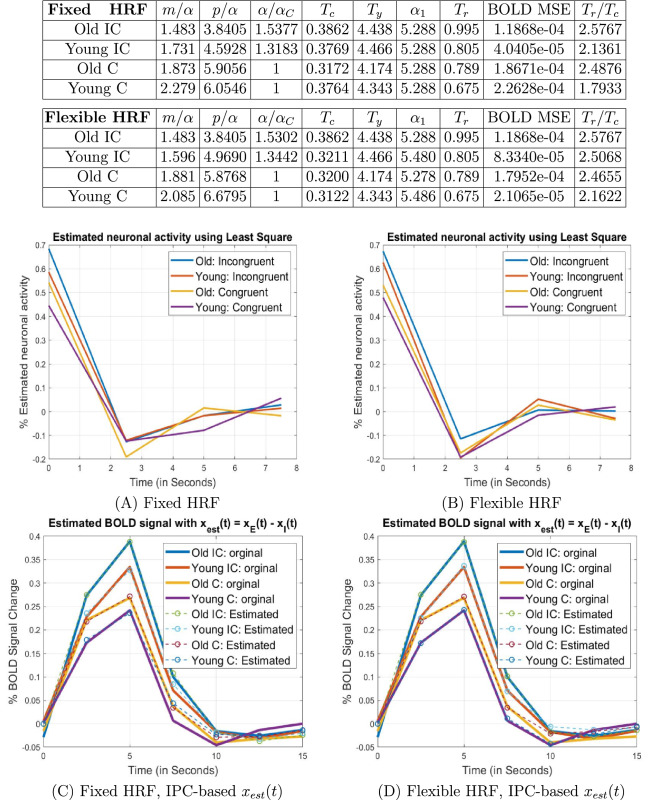
LMOG Old Analysis results for the left middle occipital gyrus (LMOG) region found in the old group. The parameter estimation results indicate that compared with the old group, the young group has higher relative information processing capacity under the same task. In (C) and (D), the estimated BOLD signal $y_{est}(t) = x_{est}(t)*h(t),$ where $x_{est}(t) = x_{E}(t) - x_{I}(t)$ was obtained based on the IPC model.

Because the flanker paradigm was an event related design, we were able to characterize evoked neuronal responses using a simple form of hemodynamic deconvolution. The analysis procedure is summarized in Table 2 and more details can be found in the Methods section and supplementary material. The results for right MFG and left MOG are provided in Figs. 2 and 3, and the results for IFG, SFG and IOG can be found in supplementary material.

**Table 2:** Model verification procedure.

The blood-oxygen-level-dependent (BOLD) signal, denoted as $y(t),$ is generally modeled as a convolution^44^ of the neuronal activity or response function $x(t)$ and a canonical hemodynamic response kernel $h(t)$ ^45–47^, that is $y(t) = x(t)*h(t)$. Our analysis was conducted in three steps:
**Step 1**: *Estimate (i.e., deconvolve) the neuronal response function* $x(t)$ *from the average BOLD signal across the trials.*
**Step 2**: *Estimate the relative information processing capacity, storage capacity, and the accompanying time constant from the ensuing neuronal response function.* Due to the simplicity of the experimental design, we approximated the information arrival rate—during Congruent and Incongruent trials—as $H(t) = \alpha_{C} \delta(t)$ and $H(t) = \alpha_{IC} \delta (t)$, respectively, and estimated the requisite parameters based on the two-state model $x(t) = x_E(t)-x_I(t),$ where $x_E(t) = \frac{\alpha }{m} e^{{ - \frac{p}{m} t}} u\left( t \right) + \frac{{\alpha_{1} }}{m} e^{{ - \frac{p}{m} \left( {t - T_{1} } \right)}} u\left( {t - T_{1} } \right)$ is excitatory activity and $x_I(t) = \frac{\beta }{m} e^{{ - \frac{p}{m} \left( {t - T_{0} } \right)}} u\left( {t - T_{0} } \right)$ is inhibitory activity.
**Step 3**: *Model validation and accuracy evaluation.* With the ensuing parameter estimates, we can obtain a model-based estimate of the neuronal activity $x_{est}(t)$ and the estimated BOLD signal $y_{est} (t) = x_{est}(t)*h(t).$ The accuracy of the IPC model can then be evaluated by comparing $y_{est}(t)$ to the empirical BOLD signals.

### Under the same cognitive task, higher information processing capacity reduces the amplitude and time constants of neuronal responses

Recall that we approximated the information arrival rate during Congruent and Incongruent trials with $H(t) = \alpha_{C} \delta (t)$ and $H(t) = \alpha_{IC} \delta (t)$, respectively. Due to the simplicity of the experimental design, we can assume that both the young and old groups have the same (encoded) input information $\alpha_{C} .$ Under this assumption, we can quantify the relative information processing capacity for young and old groups. Note that generally, the processing capability—associated with a particular cognitive task—does not change over a short time, and therefore for a given age group (either young or old group), it is reasonable to assume that the information processing capacity $p$ is a constant and does not differ between the Congruent and Incongruent conditions during the experiment.

From Figs. 2 and 3, we can see that in both right MFG and left MOG, the young group has higher processing capacity, lower neuronal activity, and a smaller time constant than the old group, under the same task condition. Similar results were obtained for IFG, SFG and IOG. In other words, our analysis suggests that *in individual brain regions, higher information processing capacity reduces neuronal activity and enables faster neuronal responses under the same cognitive task.* This is consistent with the notion that to fulfill a particular task, individuals with high processing capacities tend to exert less effort and have faster responses, i.e., are more efficient in their processing. Our results echo findings in literature where it was reported that compared with young adults, more brain activation was required for older adults to accomplish the same cognitive task^27,28^.

It is interesting to note that in regions where the younger group demonstrates significantly higher processing capacity than the old (e.g., the left and right MFG and left SFG), the young group also has significantly shorter time-to-peak in BOLD response.

### Within each age group, the Incongruent task furnishes a higher information arrival rate, and hence higher neuronal activity than the Congruent task

From the simulation results, it can also be observed that within both the young and old groups, $\alpha_{IC} /\alpha_{C} > 1,$ i.e., $\alpha_{IC} > \alpha_{C}$. That is, the Incongruent task entails a higher information arrival rate than the Congruent task. Concurrently, it can be seen that *both young and old groups have higher neuronal activity in the incongruent condition than the congruent condition.* This is consistent with the fact that the incongruent case is a more demanding (i.e., higher information load) task, and hence evokes a greater neuronal response.

### Overall brain performance evaluation

**Figure 4:**
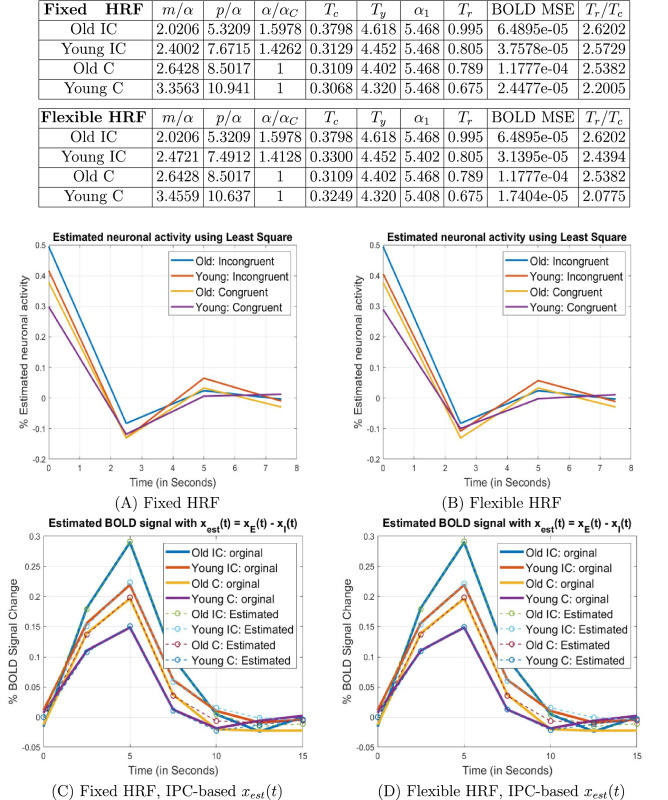
Average. Analysis results for the averaged data from all activated regions in the young and old groups. The parameter estimation results indicate that compared with the old group, the young group has higher relative information processing capacity and faster response under the same task. In (C) and (D), the estimated BOLD signal $y_{est} (t) = x_{est} (t) * h(t),$ where $x_{est}(t) = x_{E}(t) - x_{I}(t)$ was obtained based on the IPC model.

**Figure 5:**
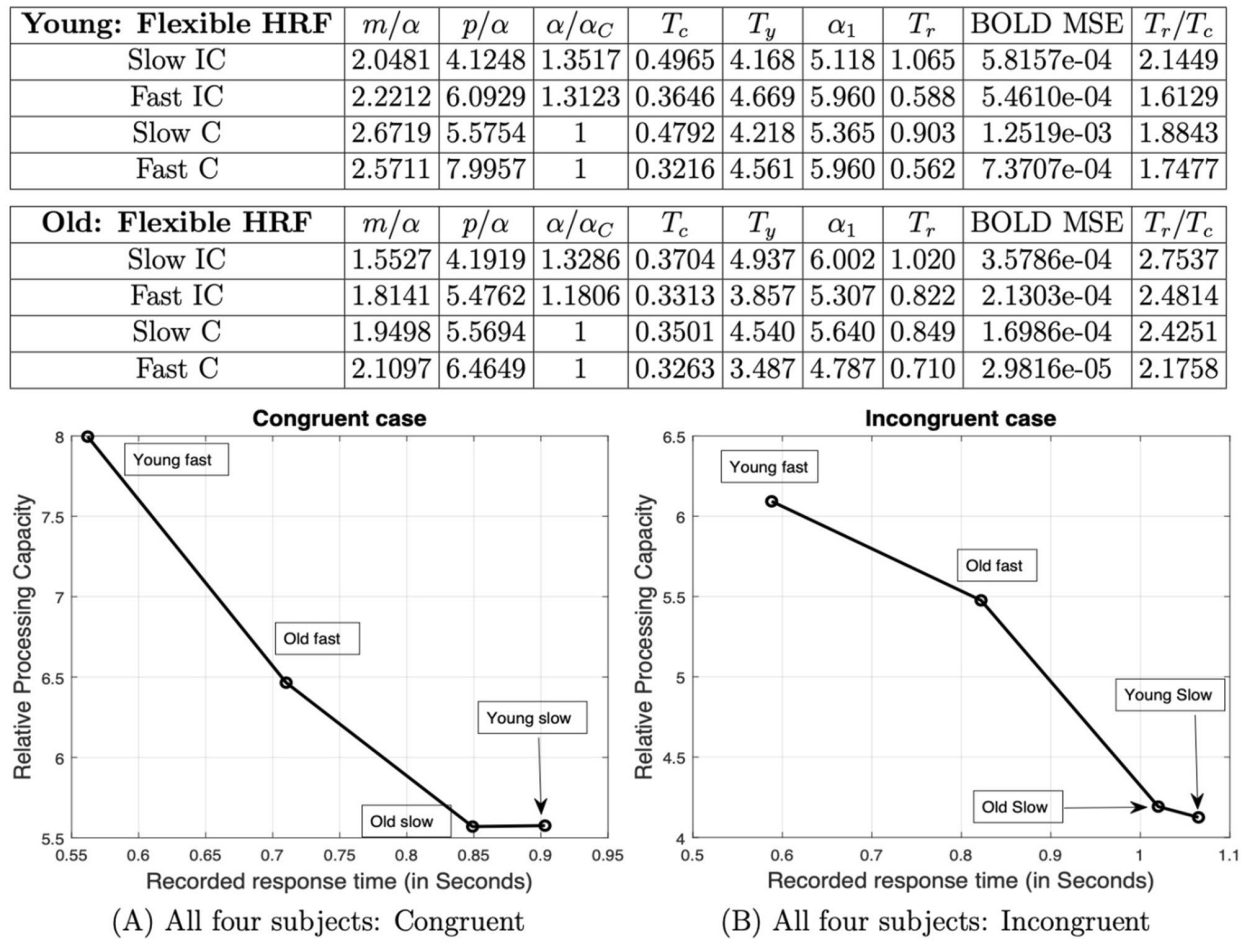
Individual difference in information processing capacity: results for the averaged BOLD across all regions for each subject. Here, relative processing capacity denotes the capacity with respect to the Congruent or Incongruent task, respectively.

To characterize overall brain performance, we averaged the data from all the active regions/clusters identified in both young and old groups and repeated the above analysis using the average responses. The result is shown in Fig. 4. We can see that, on average, compared with the older group, the younger group has higher relative information processing capacity, as well as higher relative input storage capacity and a smaller time constant (or faster response) under the same task conditions. In Fig. 5, in addition to the time constant, we have also listed the average behavioral response times for both groups under different conditions. The average time constant and the behavioral response time have exactly the same profile, in descending order: Old IC, Young IC, Old C and then Young C. The ratio between the average response time and the time constant is within the range 2.07–2.62. This is an interesting result because it speaks to the predictive validity of the model. In other words, at the whole brain and group level, we were able to predict independent behavioral responses, based purely on a modelling of hemodynamic responses.

Note that due to the simplicity of the congruent task, we have assumed that both the young and old group receive the same amount of information in bits, i.e., $\alpha_{C,old} = \alpha_{C, young} .$ However, from Fig. 4, we note that on average, $\alpha_{IC,old} > \alpha_{IC, young} .$ This effect of age is consistent with a higher complexity of the incongruent task, where older adults require more redundancy in the input information than younger adults. Higher redundancy results in more encoded information, and is considered to be a neuroprotective mechanism against aging-related cognitive decline^48,49^. In other words, the code with a length of $\alpha_{IC,old}$ bits has more redundancy than that with $\alpha_{IC, young}$ bits, if both represent the same input information.

## Discussion

The contribution of this article is two-fold. First, we have established and validated—a formal model—named the IPC model—to quantify the relationship between the regional information processing capacity and its input storage capacity, neuronal activity, and the arrival rate of the input information. Second, we applied the IPC model to fMRI data obtained from a flanker test, designed to assess age-related differences in brain activation. Our numerical analysis suggests that for a given cognitive task, higher information processing capacity leads to lower neuronal activity levels and faster responses. This observation is consistent with the findings reported in literature that high-capacity individuals generally have lower neuronal activity^8^, and that—compared with young adults—greater brain activation is required for older adults to accomplish the same cognitive processing^27,28^. The numerical results also demonstrated the accuracy of our model in fMRI signal prediction.

This paper is an initial step towards the quantitative characterization of the information processing capacity of individual brain regions. In what follows, we discuss the flexibility and scalability of the model, examine the relationship of our model with existing work, and present some possible limitations and extensions that could be explored.

### Model flexibility and scalability

Our model is flexible and scalable. First, although the IPC model was initially validated with the fMRI data, it can be applied to any data type from which neuronal responses can be estimated. Second, the model rests on the information conservation law, which is a universal principle that can be applied to any lossless unit with information processing and storing capability. Therefore, our model is not limited to individual brain regions. This explains why the information-theoretic neuronal activity model developed for localized neural population has the same first-order conductance circuit functional form that describes the membrane potential of a single neuron^13,50,51^, and the neural mass models used in DCM^22,23^. Potentially, our model can serve as a bridging framework for multiscale modeling of brain dynamics, from neuron to region, and to the whole brain. In short, the IPC model links neuronal activity to information processing capacity and provides a functional perspective on neuronal computations.

### Extension to regions with information loss

Given the experimental design and the fact that all the participants were neurotypical subjects, we assumed that there was no information loss in the brain regions under consideration. However, when the information arrival rate is higher than the processing capacity, overflow could occur. Under information loss, the original information conservation law needs to be generalized as: $c(t)I(t) = I_{p}(t) + I_{m}(t)$, where $0 \le c(t) \le 1$ denotes the portion of the information that is *not* lost by the region. If $c(t) = 1,$ it means that no information is lost in the region; if $c(t) = 0,$ it means that all the information is lost. If we assume that $c(t)$ is a constant during a short trial, then the first-order circuit IPC model in Eq. (3) can be generalized as

4 $$\frac{dx(t)}{{dt}} = - \frac{p}{m}x(t) + \frac{c}{m}H(t).$$

This generalized IPC model may help us evaluate the information loss in different neural systems or brain regions, especially those involved in overflow-driven faulty decision making or abnormal conditions such as Alzheimer’s disease or seizures.

### Similarity and differences with Shannon’s mutual information framework

A communication network, like the brain network, is a connected set of nodes/regions. The similarity between the IPC model and Shannon's mutual information framework lies in that both involves information conservation. However, Shannon's mutual information framework characterizes the communication or connectivity between nodes, while our information conservation law (and the implicit IPC model) considers information as an extensive quantity in terms of receiving, processing, and storage at each node.

More specifically, recall that in a communication system, if *X* is the signal emitted by the source and transmitted through a channel, and *Y* is the signal received at the destination, the mutual information *I(X,Y)* represents the information conveyed by the channel; in other words, the information obtained (or recovered) about *X* when *Y* is observed. The largest mutual information that can be achieved over a channel is called the capacity of the channel. Note that Shannon’s mutual information is based on a conservation law concerning information transmission between nodes:

$$\begin{aligned} The \, total \, information \, in \, X &= the \, information \, recovered \, about \, X \, after \, Y \, is \, received \, \\ & \quad \left( {i.e., \, the \, mutual \, information} \right) \, + \, the \, information \, left \, in \, X \\ & \quad \left( {i.e., \, not \, recovered} \right) \, after \, Y \, is \, received. \\ \end{aligned}$$

The information conservation law we have introduced, on the other hand, concerns the information processing and storing in individual nodes:

$$\begin{aligned} & The \, total \, information \, delivered \, to \, the \, region \\ & \quad = \, the \, information \, taken \, for \, processing \\ & \qquad + \, the \, information \, saved \, in \, the \, input \, storage \, unit. \\ \end{aligned}$$

From a communication network perspective, the information conservation law introduced here can be regarded as the counterpart to Shannon’s mutual information framework, and allows us to evaluate the information processing and storing capability of each individual region. In this way, when cognitive impairment occurs, it is possible for us to identify whether there is impaired regional processing, or connectivity, or both.

### Relationship with neural mass models

Dynamic causal modelling (DCM) generally characterizes the self-connectivity and cross-connectivity of the brain regions using a model based upon coupled neuronal masses. If we consider an individual region and absorb the cross-connectivity (i.e., inputs from other regions) into the exogenous input $e(t)$, then we obtain a DCM for a single region. More specifically, consider a region with a single neuronal state (i.e., the region can be summarized with a single neuronal activity level at any given time instant), the synaptic activity of the neuronal population in this region can be summarized with^22,23^:

5 $$\frac{dx\left( t \right)}{{dt}} = - \sigma x\left( t \right) + \beta e\left( t \right)$$

6 $$y\left( t \right) = \mathop \int \limits_{ - \infty }^{\infty } x\left( {t - \tau } \right)h\left( \tau \right)d\tau$$

where the parameter σ is referred to as the intrinsic connectivity or self-connectivity^22,23^, in the sense that how the previous neural state influences the current state, and $\beta$ denotes the strength of the afferent input.

Compare the IPC model with the single-state DCM (S-DCM) model, we can now provide an information-theoretic interpretation of the self-connectivity parameter $\sigma$. That is, $\sigma = p/m$, which is the ratio between the processing capacity *p* and the input storage capacity *m* of the region. Moreover, we can see that the parameter $\beta = { }\frac{c}{m}{ }$ is determined by both the portion of the information retained and the storage capacity of the region. The formal connection to the S-DCM makes it possible to relate the processing and storage capacities of an individual region to the neuronal activity of other regions in the network and links this information-theoretic model of neuronal activity to the extensive research using DCM.

### Detection of individual differences in processing capacity using the IPC

To examine whether the IPC model can distinguish the differences in individuals, we selected two subjects randomly from each group, i.e., one pair from the young group and one pair from the old group. Within each pair, one subject has faster response than the other (as reflected in the response times). For each subject, we applied the IPC model to all the regions that were identified to be active during the flanker test, as well as the averaged BOLD signal across all the regions. We found that for both the young and old individual pairs, the IPC model was able to demonstrate that the faster subject has higher processing capacity than the slow subject, in most brain regions (and the average across all regions). It was also observed that compared to the group average, the BOLD signals for individual subjects are much noisier. In some regions (e.g., the right inferior frontal gyrus (IFG) in the young pair), the BOLD signals were considered too noisy for further processing, suggesting that an efficient identification of the IPC model may require noise suppression, through pooling data over regions, trials, or subjects. When we put all the four subjects together, 6 possible pairs can be formulated. IPC could detect the differences between most pairs based on the averaged BOLD across all regions for each subject. However, the young slow subject and the old slow subject, who had very close and relatively large (recorded) response times, can barely be distinguished by IPC. Results corresponding to the average BOLD signals in the young and old pairs are provided in Fig. 5. Please refer to the Supplementary Material for more details.

### Possible misinterpretations

**Figure 6:**
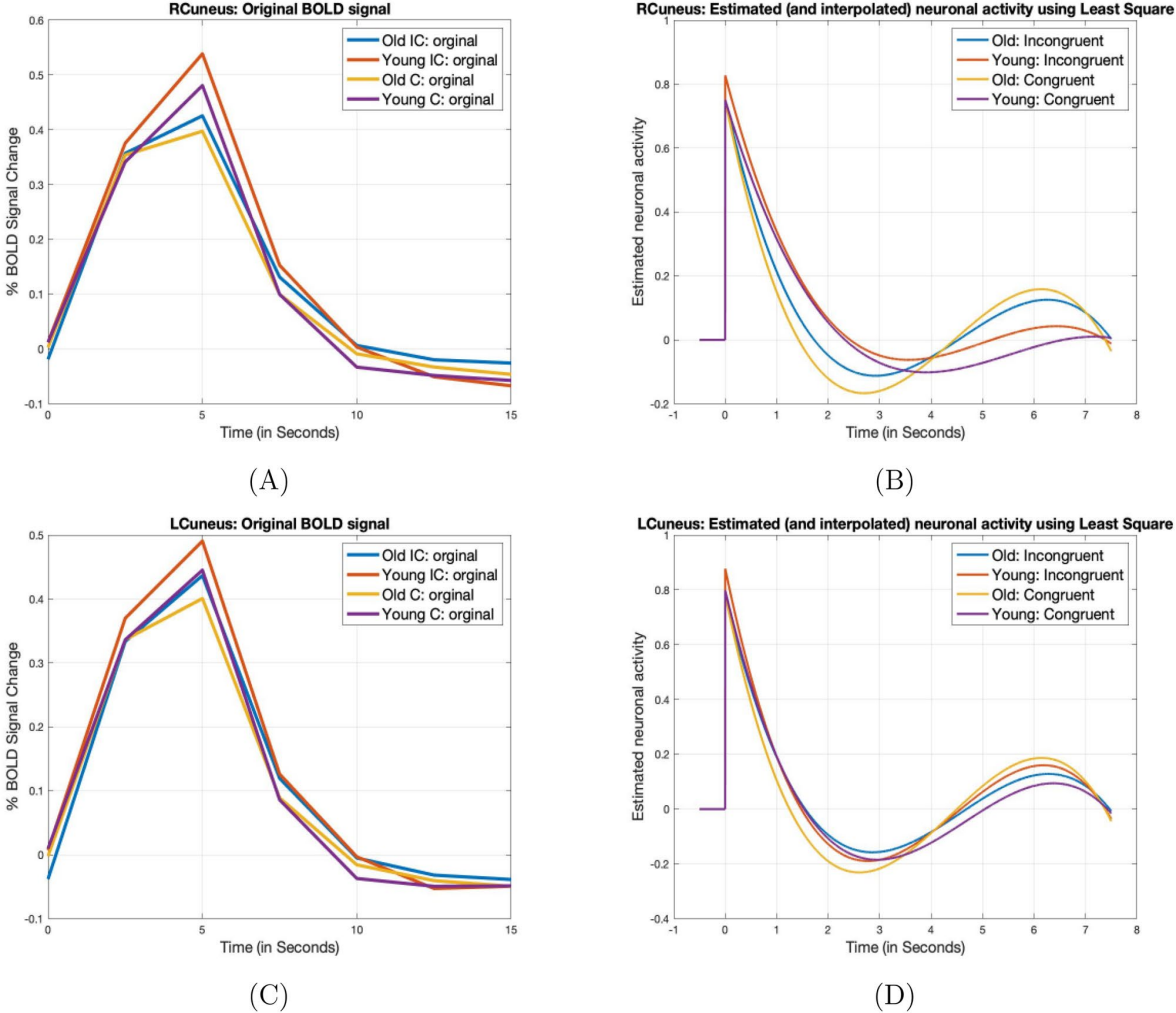
Left and Right Cunei. In these two regions, especially the right cuneus, old adults showed much lower neuronal activity than the young adults under both congruent and Incongruent conditions. This is most likely an indication of age-related brain activation decline, rather than enhanced information processing capacity.

While we say that high information processing capacity leads to lower neuronal activity, the inverse statement may not be true. One needs to proceed with care when dealing with the data of older adults, where low neuronal activity does not necessarily imply higher processing capacity but may just be a sign of age-related decline in brain activation. In this research, as shown in Fig. 6, we noticed that in the cunei (especially the right cuneus), older adults showed much lower neuronal activity than the young adults. Pending further verification, we tend to explain this as an age-related decline rather than older adults having a stronger processing capacity than the young adults in this region. Our result is consistent with the findings in^52,53^, where the age-related decline in cunei, which corresponds to the decline in visual processing efficiency, was also noted.

### Further analysis and future work

Although the IPC model demonstrated high accuracy in fMRI signal prediction, our analysis was still limited by the low sampling rate of the fMRI data, as part of our parameter estimation relied on interpolation. To improve the estimation accuracy, one might apply our analysis to data with higher time resolution, such as the EEG (especially joint EEG-fMRI^54^) and MEG data. With more precise information-theoretic parameter estimates, it will be very interesting to compare the information processing and storage capacity estimates with estimates of message passing under predictive coding models, and indeed models of Bayes-optimal decision using belief propagation. For example, one might ask how learning reduces the number of bits required for input encoding and the dimension of the decision space, hence reducing the complexity in decision making and increasing processing capacity. Further testing and refining of the model in other behavioral scenarios (e.g., working memory, attentional blink) is also a future goal of our research.

## Methods

The data used in this work was collected by David Zhu and originally used to study aging-associated decline in selective attention and executive function^42^. A full description of the subjects, image acquisition, fMRI signal pre-processing and BOLD signal extraction can be found in^42^. Here, we focus on the methods used to deconvolve the neuronal response functions from the BOLD signal, to estimate the relative information processing and storage capacities (and associated time constants) in terms of a parameterized neuronal response function. Finally, we explain how we addressed model validation and accuracy.

### Subjects

Twenty-three young adults and twenty-six older adults participated in this study. All subjects self-reported that they were free of neurological disorders. The Institutional Review Board at Michigan State University approved the study, and written informed consent was obtained from all subjects prior to the study. All methods were performed in accordance with the institution’s relevant guidelines and regulations. Data from one young subject and three older adults were discarded due to vision problems and/or highly irregular anatomical structure or a diagnosis of past stroke. Data from an additional older adult were discarded due to very low correct response rates during the flanker task (48.7% accuracy). Twenty-two young adults (11 males, age 20 ± 3 years) and twenty-two older adults (9 males, age 74 ± 6 years) were included in the data analyses. All 22 young subjects were students from Michigan State University. All 22 older subjects were recruited from Michigan State University and surrounding communities and were well educated with a mean of 16.4 (± 3.6) years of education for the 18 subjects who provided this information.

### Imaging acquisition

The experiment was conducted on a GE 3T Signa® HDx MR scanner (GE Healthcare, Waukesha, WI) with an 8-channel head coil. During each session, images were first acquired for the purpose of localization, and then first and higher-order shimming procedures were carried out to improve magnetic field homogeneity^55^. Echo planar images (EPI), starting from the most inferior regions of the brain, were then acquired with the following parameters: 34 contiguous 3-mm axial slices in an interleaved order, TE = 27.7 ms*,* TR = 2500 ms, flip angle = 80°, FOV = 22 cm, matrix size = 64 × 64, ramp sampling, and with the first four data points discarded. Each volume of slices was acquired 164 times during each of the four functional runs, while a subject viewed the stimuli and pressed a button to indicate the pointing direction of the central arrow, resulting in a total of 656 volumes of images over the course of the entire experiment. After functional data acquisition, high-resolution volumetric *T*_1_-weighted spoiled gradient recalled (SPGR) images—with cerebrospinal fluid suppressed—were obtained, covering the whole brain with 120 1.5-mm sagittal slices, 500 ms time of inversion, 8° flip angle and 24 cm FOV. These images were used to identify anatomical locations.

### Experimental paradigm for fMRI

This study employed a flanker task with a rapid event-related design, including a total of 128 trials for each of three conditions: Incongruent, Congruent and Neutral. Each of the four 7-min runs started with a 10-s baseline condition (a fixation cross) followed by stimulus trials (32 for each condition) presented in random order and with randomized inter-stimulus intervals (ISI) at multiples of 2.5 s (mean of ISI = 4.27 s). A fixation cross was presented as the baseline condition between stimuli. The stimuli for all trials—and the fixation cross—were presented in white on a black background. Each stimulus array was presented for 2.5 s, during which time the participant pressed a button to identify the direction of the central arrowhead. Subjects were naive to the flanker task but performed a 2-min practice flanker task immediately before the imaging.

The stimulus trials were randomized with the “RSFgen” program in AFNI software^43^ for optimizing the calculation of the hemodynamic response function for each stimulus condition and for the contrasts between them. For each stimulus condition, targets and flankers were presented in the two possible directions an equal number of times. Stimuli were displayed on a 640 × 480 LCD monitor mounted on top of the RF head coil. The LCD subtended 12° × 16° of visual angle. The paradigm was controlled by an IFIS-SA system (Invivo Corp., Gainesville, FL). A pair of 5-button MR-compatible keypads with this system was used to record participant responses.

### fMRI Data pre-processing

All fMRI data pre-processing was conducted with AFNI software^43^. For each subject, the acquisition timing difference was first corrected for different slice locations. With the first functional image as the reference, rigid-body motion correction was performed in three translational and three rotational directions. The amount of motion in these directions was estimated and used as a nuisance regressors in subsequent data analysis. For each subject, spatial blurring with a full-width half maximum of 4 mm was used to suppress measurement noise, and to reduce the effects of inter-subject anatomical variation (and Talairach transformation) during group analysis. For the group analysis, all images were converted to Talairach coordinate space^56^ with interpolation to 1 mm^3^ voxels. The coordinates of brain activity are presented in Talairach space in the format of (RL, AP, IS) in mm, where R = Right, L = Left, A = Anterior, P = Posterior, I = Inferior, and S = Superior.

The impulse response function (IRF) at each voxel with respect to each stimulus condition was resolved with multiple linear regression using the “3dDeconvolve” software in AFNI^57^ In the “3dDeconvolve” procedure, MRI signal modeling included subject motion regressors in the three translational and the three rotational directions, and the constant, linear and quadratic trends for each of the four functional runs. The IRFs were resolved to seven points from zero to 15 s at the resolution of 2.5 s (TR). The BOLD signal change was calculated based on the area under the IRF curve. The equivalent BOLD percent signal change relative to the baseline state was then calculated.

Although the task included the Neutral (N) condition described above, in data analysis, we focused on the Congruent (C) and Incongruent (IC) conditions since Congruent versus Incongruent comparisons yield the greatest effect sizes. Moreover, to avoid the impact of possible activation pattern differences in incorrect trials (especially in older adults), the fMRI data used for analysis in this article were based on correct (i.e., successful) trials only.

### Neuronal activity deconvolution, parameter estimation and performance evaluation

Our analysis comprised three steps:

1. *Estimating the neuronal response function* $x(t)$ *from the BOLD signal* $y(t)$ *using Least Squares deconvolution.* We assumed that: $y(t) = x(t)*h(t) + n(t),$ where $n(t)$ denotes the noise in the BOLD signal, and $h(t)$ is a canonical hemodynamic response function [45]. More specifically, $h\left( t \right) = A\left( {\frac{{t^{{{\upalpha }_{1} - 1}} {\upbeta }_{1}^{{{\upalpha }_{1} }} e^{{ - {\upbeta }_{1} t}} }}{{{\Gamma }\left( {{\upalpha }_{1} } \right)}} - c\frac{{t^{{{\upalpha }_{2} - 1}} {\upbeta }_{2}^{{{\upalpha }_{2} }} e^{{ - {\upbeta }_{2} t}} }}{{{\Gamma }\left( {{\upalpha }_{2} } \right)}}} \right),$here $\upalpha_{1}$ denotes the ratio of the response delay and response dispersion, ${\upalpha }_{2}$ denotes the ratio of the undershoot delay and undershoot dispersion, ${\upbeta }_{1}$ is the reciprocal of the response dispersion (default = 1), ${\upbeta }_{2}$ is the reciprocal of the undershoot dispersion (default = 1), *c* the ratio of undershoot to response (default = 1/6) and $A$ is the scaling parameter of the hemodynamic response function. Note that if we adopt the default values for the response dispersion and undershoot dispersion, then $\upalpha_{1}$ = the response delay, and $\upalpha_{2}$ = the undershoot delay. Analyses were carried out under both *fixed* and *flexible* HRFs. The basic idea was to select $h(t)$ to minimize/reduce the mean-square-error (MSE) between the true BOLD signal $y(t)$ and the estimated BOLD signal $y_{est}(t) = x_{est}(t) *h(t),$ where $x_{est}(t)$ was obtained for the selected $h(t)$ using the Least-Square method. For fixed HRF, $h(t)$ was selected with respect to the old group under Incongruent condition and assumed to be invariant for each region, and the same $h(t)$ was used for both young and old groups, and under both Incongruent and Congruent Conditions. For each region, the response delay and undershoot delay of the HRF were adjusted based on the experimental BOLD signals^46,47,58,59^. For Flexible HRFs, $h(t)$ was adjusted for each group (Young vs. Old) and under different conditions (Congruent vs. Incongruent). More details can be found in the supplementary material.
2. *Estimating the relative information processing capacity, input storage capacity and the system time constant from the neuronal response functions.* First, we interpolated the estimated neuronal response function $x(t)$ using a standard cubic spline interpolation function in MATLAB. Due to the simplicity of the experimental design, we approximated the information arrival rate corresponding to the Congruent and Incongruent conditions as $H(t) = \alpha_{C} \delta (t)$ and $H(t) = \alpha_{IC} \delta (t)$, respectively. Based on our framework and the observation of the estimated neuronal activity, we model the neuronal response as $x(t) = x_E(t) -x_I(t),$ where $x_E(t) = \frac{\alpha }{m} e^{{ - \frac{p}{m} t}} u\left( t \right) + \frac{{\alpha_{1} }}{m} e^{{ - \frac{p}{m} \left( {t - T_{1} } \right)}} u\left( {t - T_{1} } \right)$ is the excitatory component and $x_I(t) = \frac{\beta }{m} e^{{ - \frac{p}{m} \left( {t - T_{0} } \right)}} u\left( {t - T_{0} } \right)$ is the inhibitory component of the neuronal response. Note that for our data, the sampling period $T = 2.5s,$ we can rewrite the inhibitory component as $x_I(t) = b e^{{ - \frac{p}{m} \left( {t - T} \right)}} u\left( {t - T_{0} } \right)$ and the excitatory component as $x_E(t) = \frac{\alpha }{m} e^{{ - \frac{p}{m} t}} u\left( t \right) + c e^{{ - \frac{p}{m} \left( {t - 2T} \right)}} u\left( {t - T_{1} } \right).$ This fitting procedure provides estimates of $\frac{\alpha }{m}, \frac{p}{m}, b, c,T_{0} , T_{1}$ and hence the relative information processing capacity $\frac{p}{\alpha } ,$ the relative input storage capacity $\frac{m}{\alpha } ,$ and the system time constant $T_{c} = \frac{m}{p} .$ The details are provided in supplementary material.
3. *Valuation of accuracy.* For model validation and accuracy evaluation, using the estimated $\frac{\alpha }{m}, \frac{p}{m}, b, c,T_{0} , T_{1, }$ we predicted the excitatory activity $x_{E}(t),$ and the inhibitory activity $x_{I}(t)$, and hence obtained an estimate of the overall neuronal activity $x_{est}(t) = x_{E}(t) - x_{I}(t).$ We then predict the BOLD signal with $y_{est}(t) = x_{est}(t)*h(t),$ and evaluate the accuracy or model fit by comparing the predicted BOLD signal and the empirical BOLD signal obtained using fMRI.

## Supplementary Information

Supplementary Information.

## Data Availability

All the simulation data used in this research are available in the main text or the supplementary materials. Access to the experimental fMRI data is subject to standard material transfer agreements.
