# Publisher Correction: Brain information processing capacity modelling

**DOI:** 10.1038/s41598-022-07499-4

**Published:** 2022-02-25

**Authors:** Tongtong Li, Yu Zheng, Zhe Wang, David C. Zhu, Jian Ren, Taosheng Liu, Karl Friston

**Affiliations:** 1 Department of Electrical and Computer Engineering, Michigan State University, East Lansing, MI 48824 USA; 2 Department of Radiology, Michigan State University, East Lansing, MI 48824 USA; 3 Department of Psychology, Michigan State University, East Lansing, MI 48824 USA; 4 The Wellcome Centre for Human Neuroimaging, University College London, London, UK

Correction to: *Scientific Reports* 10.1038/s41598-022-05870-z, published online 09 February 2022

The original version of this Article contained an error in the spelling of the author Taosheng Liu which was incorrectly given as Taosheng S. Liu.

The original Article has been corrected.

