# Supplementary material for: Brain information processing capacity modeling
Source: Sci Rep. 2022 Feb 9;12:2174. doi: 10.1038/s41598-022-05870-z (PMC8828878; doi:10.1038/s41598-022-05870-z)
Supplement: Supplementary file 1 — Supplementary Information. [file 41598_2022_5870_MOESM1_ESM.pdf]

Suppplementary Material

This document has three Sections. In Section I, we presented the detailed techniques used in hemodynamic response function generation/selection, neuronal activity extraction and parameter estimation. In Section II, we provided the simulation results based on the group averages of the BOLD signals, and also reproduced the average response time and accuracy of the young and old groups recorded in the flanker experiment [1]. In Section III, we presented the simulation results for individual subjects pairs—one pair from the Young group and one pair from the Old group.

I. Signal and Parameter Estimation

A. Fixed and Flexible Hemodynamic Response Functions

We conducted the simulation with flexible HRFs, and got similar results as using fixed HRF, i.e., people with higher processing capacity generally have lower neuronal activity (or effort level) and faster response. Compared with fixed HRF, flexible HRFs tend to deliver better results in the sense that they can slightly reduce the MSE between the true BOLD signal and the estimated version using the IPC model; though for regions where the young and old have close time to peak in the BOLD signals, the improvement is not very significant.

It was also observed that in some brain regions (such as the left superior frontal gyrus (LSFG) cluster found only in the old group, and the right middle frontal gyrus (RMFG) found in both the young and old groups), the time to peak in the BOLD response and the response delay of the estimated HRF of the older group are larger than that of the young group, while in other regions the differences are not significant.

In the simulation, we started with the well-accepted model $y(t) = h(t) * x(t) + n(t)$, where $y(t)$ is the BOLD signal, $x(t)$ is the neuronal activity, $h(t)$ the HRF and $n(t)$ the noise term. Estimating HRF from the BOLD signal is a challenging task due to the inherent ambiguity in the model, since both the HRF $h(t)$ and the neuronal activity $x(t)$ are actually unknown, though $x(t)$ is often approximated as an impulse response under impulse like stimuli. In other words, changes in the BOLD response can either be caused by the changes in HRF or neuronal activity. In our simulation, we used the canonical HRF generated using SPM,

$$h(t) = A\left(\frac{\beta_1^{\alpha_1} t^{\alpha_1 - 1} e^{-\beta_1 t}}{\Gamma(\alpha_1)} - c\frac{\beta_2^{\alpha_2} t^{\alpha_2 - 1} e^{-\beta_2 t}}{\Gamma(\alpha_2)}\right), \tag{1}$$

where α_1 denotes the ratio of the response delay and response dispersion, α_2 denotes the ratio of the undershoot delay and undershoot dispersion, β_1 is the reciprocal of the response dispersion (default = 1), β_2 is the reciprocal of the undershoot dispersion (default = 1), c is the ratio of the undershoot to response (default = 1/6), and A is the scaling parameter of the hemodynamic response function. We adopted the default value for β_1, β_2 and c, and set $A = 1.25$ to ensure that the neuronal activity $x(t) \leq 1$ (since $x(t)$ represents the activity/effort level of the localized neuron populations in the IPC model). Note that if we adopt the default values for the response dispersion and undershoot dispersion, then $\alpha_1 =$ the response delay, and $\alpha_2 =$ the undershoot delay. We found that compared to the undershoot delay, the response delay has much more significant impact on the results, as the undershoot has a much smaller amplitude than the response. With a given HRF, say $h(t)$, we can estimate $x(t)$ from the BOLD signal $y(t)$ using the Least-Square method and obtain $x_{est}(t)$. We can then get an estimated BOLD signal $y_{est}(t) = h(t) * x_{est}(t)$, and compare that with the original BOLD signal $y(t)$. Although both $h(t)$ and $x(t)$ are unknown, it was observed that if the response delay is not selected appropriately, and there would be a big gap between the estimated and the true BOLD signals, as shown in Figure 1.

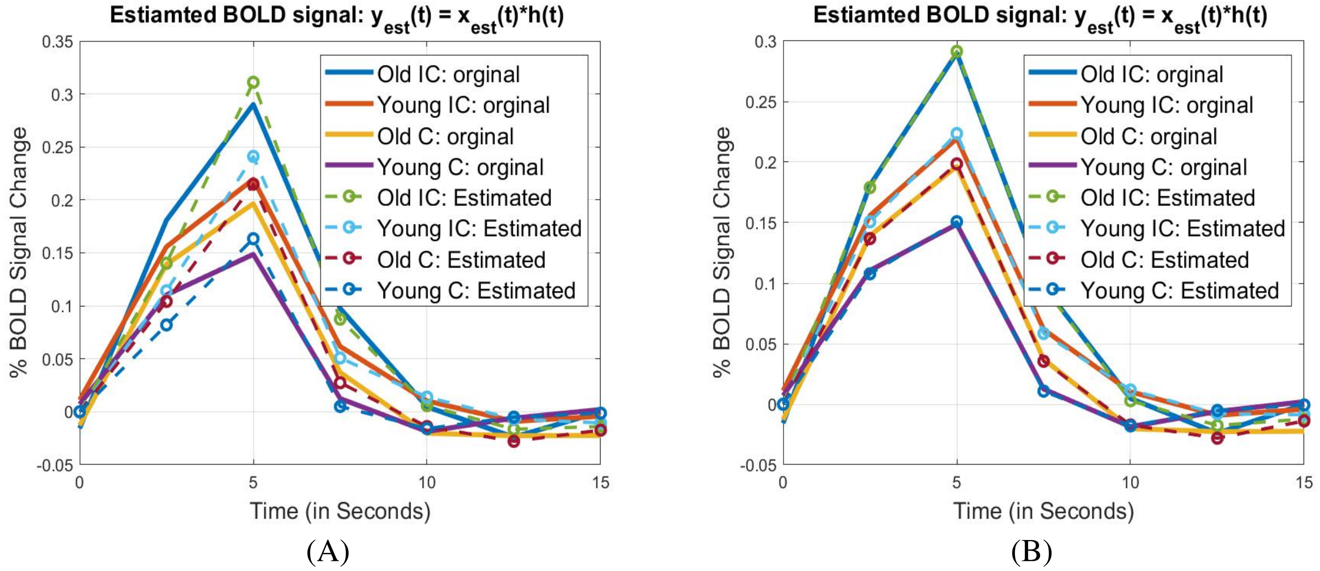

Fig. 1: Analysis for the averaged BOLD signals across all activated regions in the young and old groups—original BOLD signals versus the estimated BOLD signals under fixed HRF with different response delays. (A) Response delay $\alpha_1 = 6.00$ seconds; (B) Response delay $\alpha_1 = 5.47$ seconds;

TABLE I: MSE between the true and estimated BOLD signals under fixed HRF but with different response delays.

**Condition**	$\alpha_1 = 6s$	$\alpha_1 = 5.60s$	$\alpha_1 = 5.47s$	$\alpha_1 = 5.35s$	$\alpha_1 = 5.30s$
Old IC	3.839e-04	8.928e-05	6.378e-05	6.721e-05	8.790e-05
Young IC	3.571e-04	6.477e-05	2.890e-05	2.019e-05	3.031e-05
Old C	2.734e-04	5.974e-05	4.118e-05	4.139e-05	5.692e-05
Young C	1.642e-04	2.453e-05	1.042e-05	8.450e-06	1.415e-05

We calculated the MSE between the estimated and true BOLD signals under HRF with different response delays, and the results were shown in Table I. This Table indicated that: (i) An HRF with a more suitable response delay can reduce the BOLD MSE. *This suggests that it is better to adopt flexible HRFs*; (ii) As long as the response delay of the HRF is within a certain range, reasonable BOLD MSEs (generally 10^{-4} or smaller) can be achieved. *This explains why we obtained similar results under fixed and flexible HRFs.*

In the simulation, we initiated the response delay with a value that can achieve a reasonable BOLD MSE, generally chosen to be $\alpha_1(0)$ = the time to peak of the BOLD response + ϵ, with $\epsilon \in [0.85, 1]$, and then searched over its vicinity for the (sub)optimal response delay that would minimize the BOLD MSE. This would ensure that our estimated $x(t)$ is reliable for further analysis. Sometimes, small adjustments were made so that similar (or same) HRFs were used for the same group/subject under Congruent and Incongruent conditions. We then estimated the relative information processing capacity, storage capacity and the brain circuit time constant.

To evaluate the performance of the model, we reconstructed the neuronal activitiy from the estimated information processing capacity, storage capacity and the brain circuit time constant and obtained a new $x_{est}(t) = x_E(t) - x_I(t)$, and calculated $y_{est}(t) = h(t) * x_{est}(t) = h(t) * (x_E(t) - x_I(t))$, and then evaluated the MSE between this new $y_{est}(t)$ and the original BOLD signal $y(t)$. In Sections II, simulation results based on the group averages of the BOLD signals are provided under both fixed and flexible HRFs.

B. Estimating Neuronal Activity from the BOLD Signal using Deconvolution with the Least Squar Method

Let $y(t) = x(t) * h(t) + n(t)$, where $y(t)$ is the BOLD signal percentage change, $x(t)$ the neuronal activity or the neuronal response function, $h(t)$ the hemodynamic response function (HRF) and $n(t)$ the additive noise. In the discrete-time case, we have

$$y(n) = x(n) * h(n) = \sum_{l=0}^{L} h(l)x(n-l). \tag{2}$$

Define

$$\hat{y}(n) = \sum_{k=0}^{K-1} \hat{x}(k)h(n-k), \tag{3}$$

where $\hat{x}(k)$ denotes the neuronal activity samples to be estimated. Note that $\{h(n)\}$ is a causal sequence, we can rewrite equation (2) in matrix format as:

$$\begin{bmatrix} \hat{y}(0) \\ \hat{y}(1) \\ \vdots \\ \hat{y}(N-1) \end{bmatrix} = \underbrace{\begin{bmatrix} h(0) & 0 & \cdots & 0 \\ h(1) & h(0) & \cdots & 0 \\ \vdots & \vdots & & \vdots \\ h(N-1) & h(N-2) & \cdots & h(N-k) \end{bmatrix}}_{H} \underbrace{\begin{bmatrix} \hat{x}(0) \\ \hat{x}(1) \\ \vdots \\ \hat{x}(K-1) \end{bmatrix}}_{\hat{\mathbf{x}}}$$

Let $\mathbf{y} = [y(0), y(1), ...y(N-1)]^T$ and $\hat{\mathbf{y}} = [\hat{y}(0), \hat{y}(1), ...\hat{y}(N-1)]^T$. With the Least Square method, we need to find $\hat{\mathbf{x}} = [\hat{x}(0), \hat{x}(1), ...\hat{x}(K-1)]^T$ to minimize the Euclidean norm of $\mathbf{y} - \hat{\mathbf{y}}$. That is, we need to find $\hat{\mathbf{x}}$ to minimize the cost function

$$J = \sum_{n=0}^{N-1} |y(n) - \hat{y}(n)|^2. \tag{4}$$

consider $\frac{\partial J}{\partial \hat{x}} = 0$, we get

$$\hat{\mathbf{x}} = \left(H^H H\right)^{-1} H^H \hat{\mathbf{y}}, \tag{5}$$

where H^H is the Hermitian matrix of H.

During the simulation, the hemodynamic response kernel $h(t)$ is generated with the standard SPM function in Matlab [2], where the response delay and undershoot delay were slightly adjusted from the default values for each individual region based on the peak instant and the successive zero crossing of the BOLD signals [3], [4].

C. Neuronal Activty Model and Parameter Estimation

In the simulation, we used the following neuronal response function for parameter estimation:

$$x(t) = \frac{\alpha}{m} e^{-\frac{p}{m}t} u(t) - \frac{\beta}{m} e^{-\frac{p}{m}(t-T_0)} u(t-T_0) + \frac{\gamma}{m} e^{-\frac{p}{m}(t-T_1)} u(t-T_1), \tag{6}$$

which is the superposition of the excitatory activity $x_E(t) = \frac{\alpha}{m} e^{-\frac{p}{m}t} u(t) + \frac{\gamma}{m} e^{-\frac{p}{m}(t-T_1)} u(t-T_1)$ and the inhibitory activity $x_I(t) = \frac{\beta}{m} e^{-\frac{p}{m}(t-T_0)} u(t-T_0)$. Here $T_1 > T_0 > 0$.

Note that the sampling period of our BOLD signal is $T = 2.5s$. With the fMRI data we worked on, in general, $(k_0 - 1)T < T_0 < k_0 T$ (with $k_0 = 1$ or $k_0 = 2$); and $(k-1)T < T_1 < kT$, with $k = 2$ or

$k = 3$. Write $t - T_0 = t - k_0T + k_0T - T_0$, $t - T_1 = t - kT + kT - T_1$, and let $\tau = \frac{m}{p}$. To maximize the accuracy of estimation, we can rewrite the neuronal activity model in (5) as:

$$x(t) = ae^{-\frac{t}{\tau}}u(t) - be^{-\frac{t-k_0T}{\tau}}u(t - T_0) + ce^{-\frac{t-kT}{\tau}}u(t - T_1), \tag{7}$$

where $a = \frac{\alpha}{m}$, $b = \frac{\beta}{m}e^{-\frac{k_0T-T_0}{\tau}}$, and $c = \frac{\gamma}{m}e^{-\frac{kT-T_1}{\tau}}$. This would allow us to estimate the parameters based on the samples of $x(t)$ at the integer multiples of T, and hence minimizes the dependence on interpolation.

Define $x_1(t) = ae^{-\frac{t}{\tau}}u(t)$, and $x_2(t) = ae^{-\frac{t}{\tau}}u(t) - be^{-\frac{t-k_0T}{\tau}}u(t - T_0)$, then it follows from equation (7) that

$$T_0 = \min\{t|t > 0 \ and \ x(t) < x_1(t)\}, \quad T_1 = \min\{t|t > T_0 \ and \ x(t) > x_2(t)\} \tag{8}$$

In the simulation, limited by the low time resolution of the BOLD signal, we interpolate the neuronal response function first before the estimation. Taking the physical meaning of T_0 and T_1 into consideration, it was observed that $\hat{T}_0$ and $\hat{T}_1$ can be roughly estimated as the first and second zero-crossings of the interpolated neuronal activity $x(t)$ after the peak instant. With equation (7), the offset in the estimates of T_0 and T_1 will not have significant impact on the estimation of parameters a, b, c, τ since $u(t)$ is the unit step function. We can estimate these parameters first, and then refine the estimates of T_0 and T_1 according to (8).

we now estimate the parameters a, b, c, τ following the steps below.

1) First, we have
$$\hat{a} = \hat{x}(0). \tag{9}$$
The **relative storage capacity** $\frac{m}{\alpha}$ can then be estimated as $\frac{\hat{m}}{\alpha} = \frac{1}{\hat{x}(0)}$.

2) Take a $t_0 < \hat{T}_0$, and note that $x(t_0) = \frac{\alpha}{m}e^{-\frac{t_0}{\tau}}$, then the **time constant** τ can be estimated as
$$\hat{\tau} = -\frac{t_0}{\ln\left[\hat{x}(t_0)/\hat{x}(0)\right]}, \tag{10}$$
and **the relative information processing capacity** $\frac{p}{\alpha}$ can be obtained from $\frac{p}{\alpha} = \frac{\frac{m}{\alpha}}{\tau}$, and estimated as $\frac{\hat{p}}{\alpha} = \frac{1}{\hat{\tau}\hat{x}(0)}$.

3) If $\hat{T}_0 \le T$, that is $k_0 = 1$, then from $x(T) = ae^{-\frac{T}{\tau}} - b$, we can get
$$\hat{b} = \hat{a}e^{-\frac{T}{\hat{\tau}}} - \hat{x}(T) \tag{11}$$
Note that $\hat{a}e^{-\frac{T}{\tau}}$ is essentially 0, we have $\hat{b} \approx -\hat{x}(T)$.
If $T < \hat{T}_0 \le 2T$, that is $k_0 = 2$, then from $x(2T) = ae^{-\frac{2T}{\tau}} - b$, we can get
$$\hat{b} = \hat{a}e^{-\frac{2T}{\hat{\tau}}} - \hat{x}(2T) \approx -\hat{x}(2T). \tag{12}$$

4) Finally, if $T < \hat{T}_1 < 2T$, that is, $k = 2$, then from $x(2T) = ae^{-\frac{2T}{\tau}} - be^{-\frac{T}{\tau}} + c$, we can get
$$\hat{c} = \hat{x}(2T) - \hat{a}e^{-\frac{2T}{\hat{\tau}}} + \hat{b}e^{-\frac{T}{\hat{\tau}}} \approx \hat{x}(2T). \tag{13}$$
If $2T < \hat{T}_1 < 3T$, that is, $k = 3$, then from $x(3T) = ae^{-\frac{3T}{\tau}} - be^{-\frac{2T}{\tau}} + c$, we can get
$$\hat{c} = \hat{x}(3T) - \hat{a}e^{-\frac{3T}{\hat{\tau}}} + \hat{b}e^{-\frac{2T}{\hat{\tau}}} \approx \hat{x}(3T). \tag{14}$$

With all the parameters estimated, we can then obtain a model-based estimate of the neuronal activity or neuronal response function $x_{est}(t)$ from equation (7).

II. Simulation Results based on the Group Averages of the BOLD Signals

In this subsection, we provide the simulation results based on the group average for the following regions:

- The average of all the active regions.
- The right middle frontal gyrus (RMFG) cluster found from the young group.
- The left middle frontal gyrus (LMFG) cluster found from the young group.
- The right middle frontal gyrus (RMFG) cluster found from the old group.
- The left middle frontal gyrus (LMFG) cluster found from the old group.
- The left middle occipital gyrus (LMOG) cluster found from the old group.
- The right inferior frontal gyrus (RIFG) cluster found in the old group.
- The left superior frontal gyrus (LSFG) cluster found only in the old group.
- The right inferior occipital gyrus (RIOG) cluster found in the young group.
- The right inferior occipital gyrus (RIOG) cluster found in the old group.

In all the figures in this section, $x_{E1}(t) = \frac{\alpha}{m} e^{-\frac{p}{m}t} u(t)$ denote the primary excitatory neuronal response function corresponding to the input stimulus, and $x_{est}(t) = x_E(t) - x_I(t)$ denote the estimate of the overall neuronal response function that includes both the excitatory and inhibitory neuronal activities as shown in equation (7). As demonstrated in the simulation results, in all the four regions we can get much more accurate estimation with $x_{est}(t) = x_E(t) - x_I(t)$ than that with $x_{est} = x_{E1}(t)$ only.

Our simulation results indicated that in most regions, compared with the older group, the young group has higher relative information processing capacity, lower neuronal activity, and a smaller time constant (or faster response) under the same task. In the right inferior occipital gyrus (RIOG) cluster found from the young group, the young and old seem to have comparable information processing capacity.

At the end of this section, we also provided behavioral analysis results reporduced from [1].

A. *Region name: All Average*

**Fixed HRF**	m/α	p/α	α/α_C	T_c	T_y	α_1	T_r	BOLD MSE	T_r/T_c
Old IC	2.0206	5.3209	1.5978	0.3798	4.618	5.468	0.995	6.4895e-05	2.6202
Young IC	2.4002	7.6715	1.4262	0.3129	4.452	5.468	0.805	3.7578e-05	2.5729
Old C	2.6428	8.5017	1	0.3109	4.402	5.468	0.789	1.1777e-04	2.5382
Young C	3.3563	10.9410	1	0.3068	4.320	5.468	0.675	2.4477e-05	2.2005

**Flexible HRF**	m/α	p/α	α/α_C	T_c	T_y	α_1	T_r	BOLD MSE	T_r/T_c
Old IC	2.0206	5.3209	1.5978	0.3798	4.618	5.468	0.995	6.4895e-05	2.6202
Young IC	2.4721	7.4912	1.4128	0.3300	4.452	5.402	0.805	3.1395e-05	2.4394
Old C	2.6428	8.5017	1	0.3109	4.402	5.468	0.789	1.1777e-04	2.5382
Young C	3.4559	10.6370	1	0.3249	4.320	5.408	0.675	1.7404e-05	2.0775

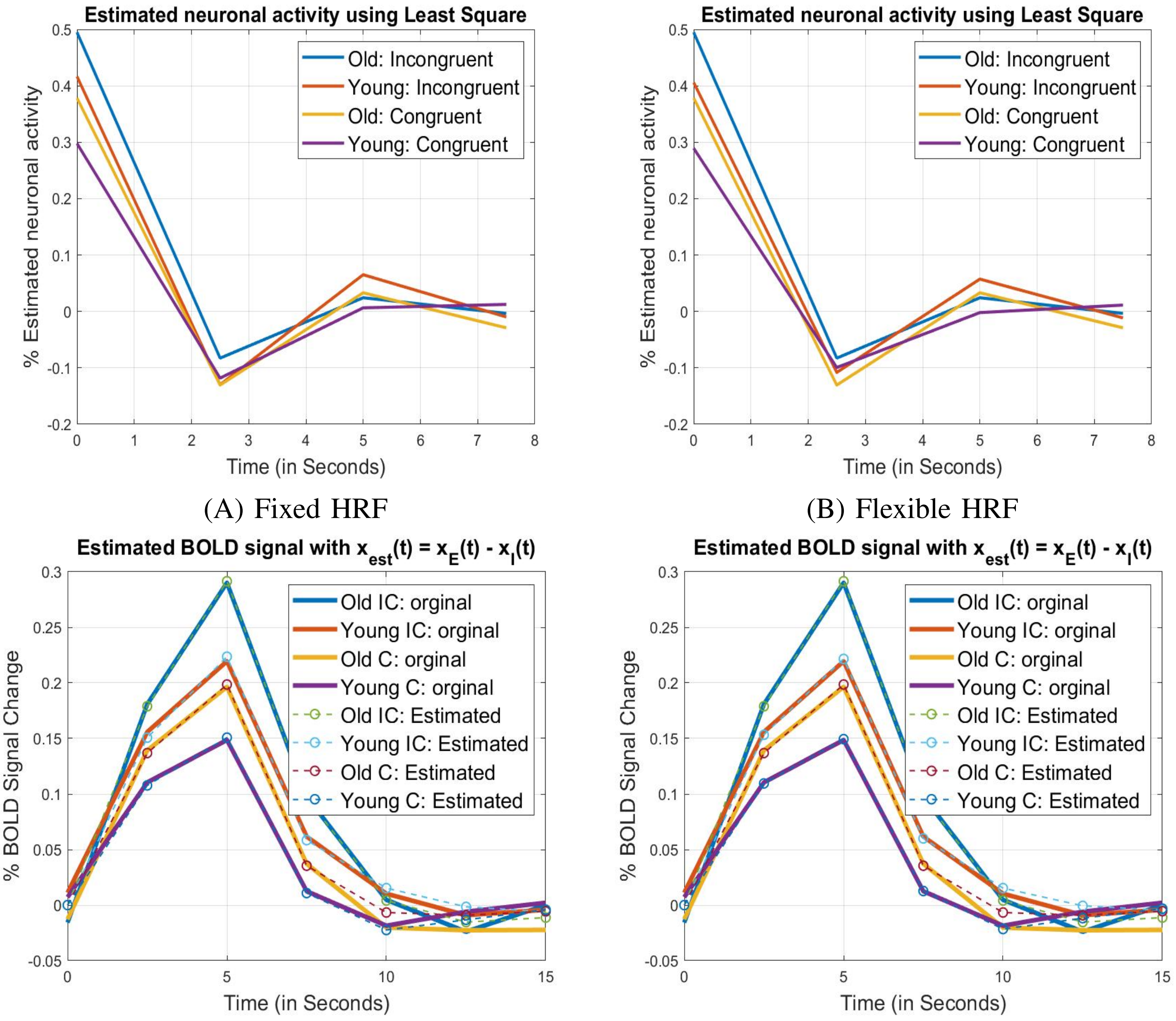

(A) Fixed HRF (B) Flexible HRF

(C) Fixed HRF, IPC-based $x_{est}(t)$ (D) Flexible HRF, IPC-based $x_{est}(t)$

Fig. 2: The averaged data across all activated regions in the young and old groups

B. Region name: Right MFG Found in the Young Group

**Fixed HRF**	m/α	p/α	α/α_C	T_c	T_y	α_1	T_r	BOLD MSE	T_r/T_c
Old IC	3.342	8.6113	1.4825	0.3881	4.332	5.182	0.995	1.4061e-05	2.5638
Young IC	4.1434	13.562	1.786	0.3055	4.004	5.182	0.805	3.1135e-05	2.6350
Old C	4.4860	12.767	1	0.3514	4.254	5.182	0.789	2.0030e-05	2.2454
Young C	6.3775	24.223	1	0.2633	3.541	5.182	0.675	4.9074e-06	2.5638

**Flexible HRF**	m/α	p/α	α/α_C	T_c	T_y	α_1	T_r	BOLD MSE	T_r/T_c
Old IC	3.342	8.6113	1.4825	0.3881	4.332	5.182	0.995	1.4061e-05	2.5638
Young IC	4.2441	12.494	1.7058	0.3397	4.004	5.154	0.805	1.4145e-05	2.3699
Old C	4.4860	12.767	1	0.3514	4.254	5.182	0.789	2.0030e-05	2.2454
Young C	6.9012	21.313	1	0.3238	3.541	5.02	0.675	1.5689e-06	2.0846

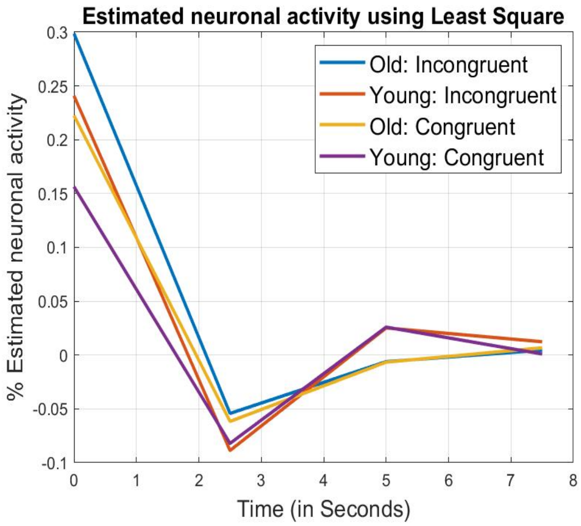

(A) Fixed HRF

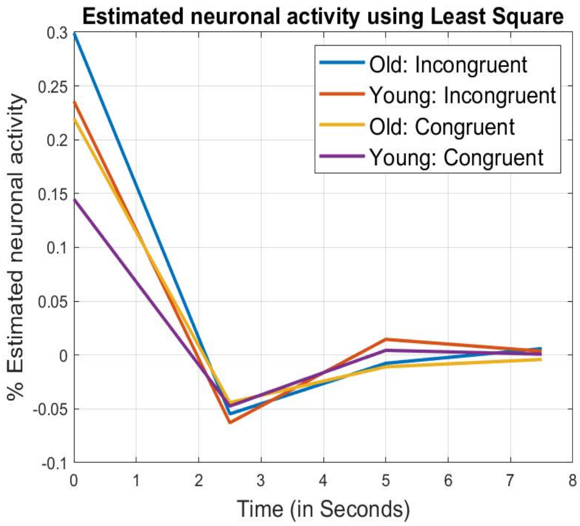

(B) Flexible HRF

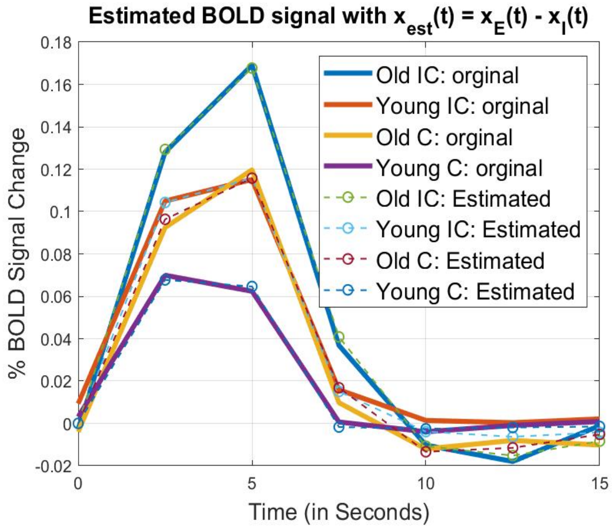

(C) Fixed HRF, IPC-based $x_{est}(t)$

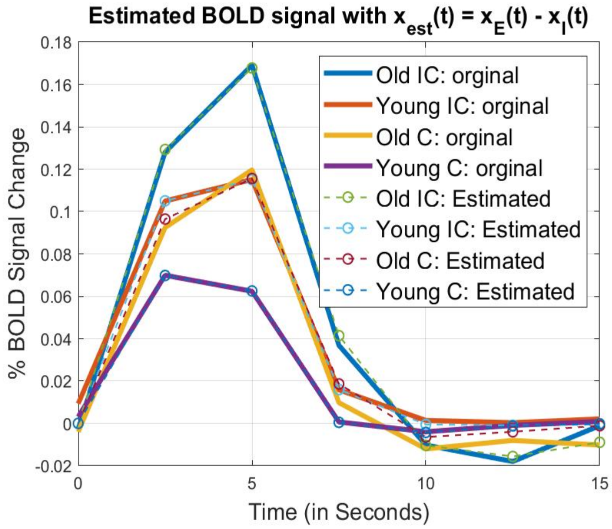

(D) Flexible HRF, IPC-based $x_{est}(t)$

Fig. 3: Results for the right middle frontal gyrus (RMFG) found in the young group.

C. Region name: Left MFG Found in the Young Group

**Fixed HRF**	m/α	p/α	α/α_C	T_c	T_y	α_1	T_r	BOLD MSE	T_r/T_c
Old IC	3.3976	6.996	1.6066	0.4857	3.815	4.665	0.995	1.0043e-04	2.0488
Young IC	4.0109	13.645	1.5711	0.2939	2.678	4.665	0.805	1.7450e-05	2.7386
Old C	3.8982	11.24	1	0.3468	3.165	4.665	0.789	9.5036e-05	2.275
Young C	5.9352	21.438	1	0.2769	2.384	4.665	0.675	5.3459e-05	2.4381

**Flexible HRF**	m/α	p/α	α/α_C	T_c	T_y	α_1	T_r	BOLD MSE	T_r/T_c
Old IC	3.3976	6.996	1.6066	0.4857	3.815	4.665	0.995	1.0043e-04	2.0488
Young IC	3.9858	13.654	1.403	0.2919	2.678	4.678	0.805	1.7074e-05	2.7576
Old C	3.9989	10.999	1	0.3636	3.165	4.615	0.789	8.8113e-05	2.1702
Young C	5.874	19.156	1	0.3066	2.384	4.634	0.675	4.9676e-05	2.2013

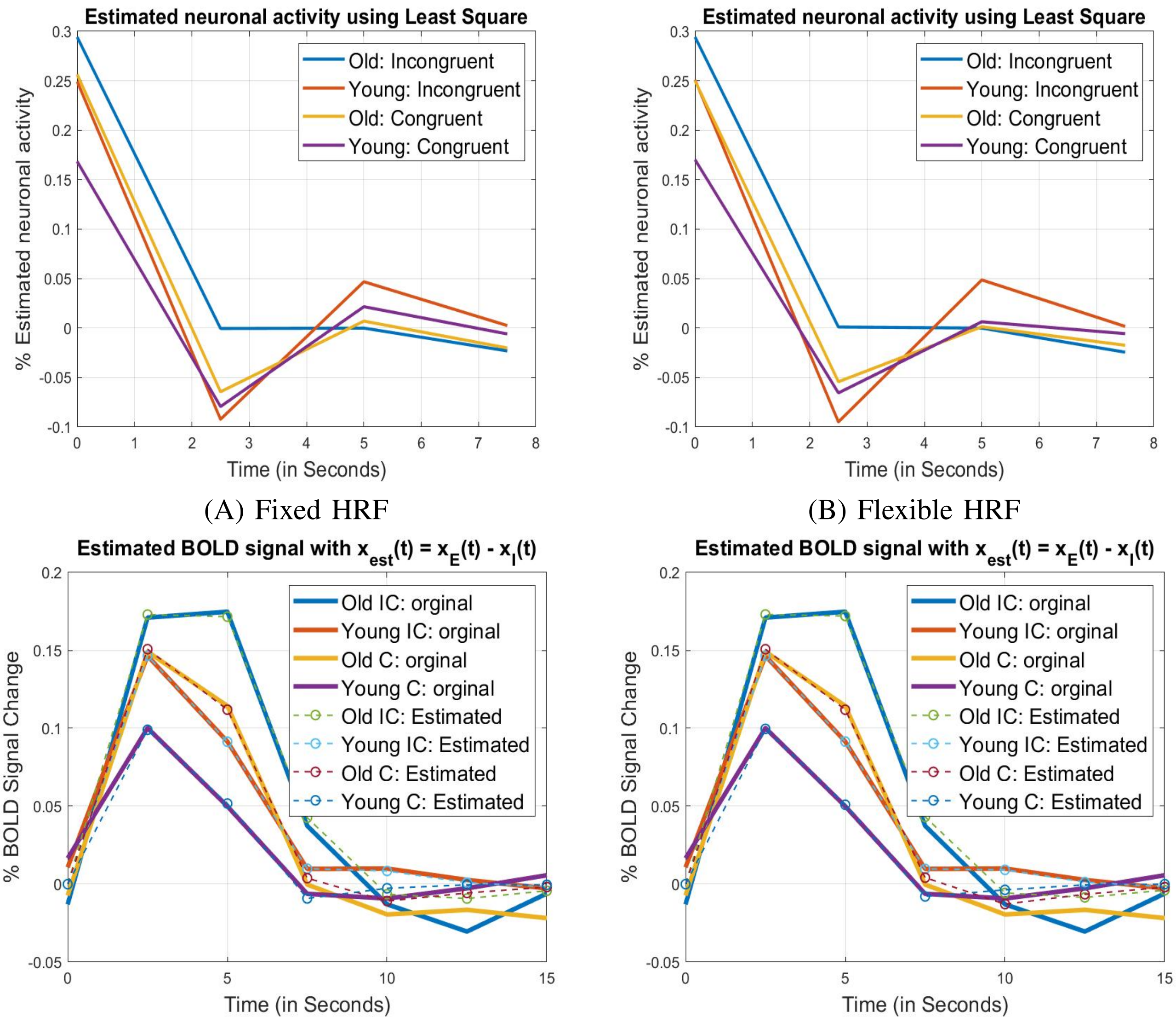

(A) Fixed HRF (B) Flexible HRF

(C) Fixed HRF, IPC-based $x_{est}(t)$ (D) Flexible HRF, IPC-based $x_{est}(t)$

Fig. 4: Results for the left middle frontal gyrus (LMFG) found in the young group.

D. Region name: Right MFG Found in the Old Group

**Fixed HRF**	m/α	p/α	α/α_C	T_c	T_y	α_1	T_r	BOLD MSE	T_r/T_c
Old IC	2.5391	7.2807	1.5646	0.34875	4.368	5.218	0.995	1.8109e-04	2.8531
Young IC	4.0753	14.163	1.9409	0.28774	4.07	5.218	0.805	2.8036e-05	2.7976
Old C	3.5106	11.391	1	0.30819	4.102	5.218	0.789	1.6469e-04	2.5601
Young C	6.98	27.488	1	0.25393	3.24	5.218	0.675	6.7804e-05	2.6583

**Flexible HRF**	m/α	p/α	α/α_C	T_c	T_y	α_1	T_r	BOLD MSE	T_r/T_c
Old IC	2.5391	7.2807	1.5646	0.34875	4.368	5.218	0.995	1.8109e-04	2.8531
Young IC	4.09	14.173	1.9008	0.28856	4.07	5.21	0.805	2.7976e-05	2.7897
Old C	3.7224	10.799	1	0.34471	4.102	5.102	0.789	1.5975e-04	2.2889
Young C	7.296	26.941	1	0.27082	3.24	5.09	0.675	6.452e-05	2.4925

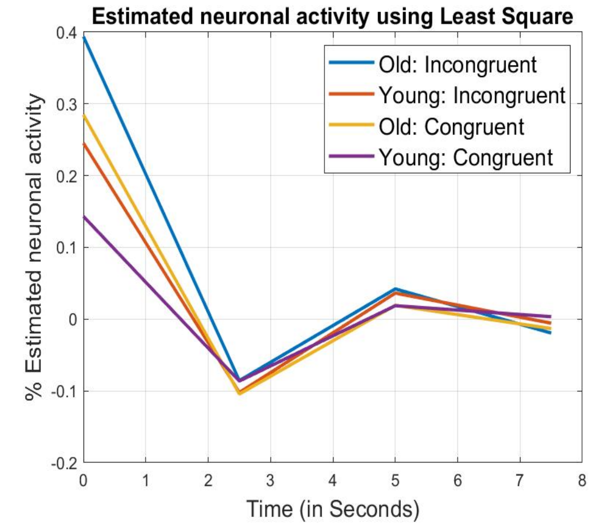

(A) Fixed HRF

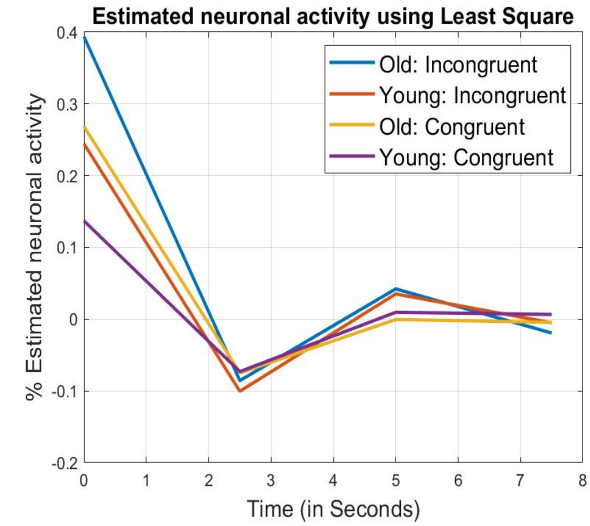

(B) Flexible HRF

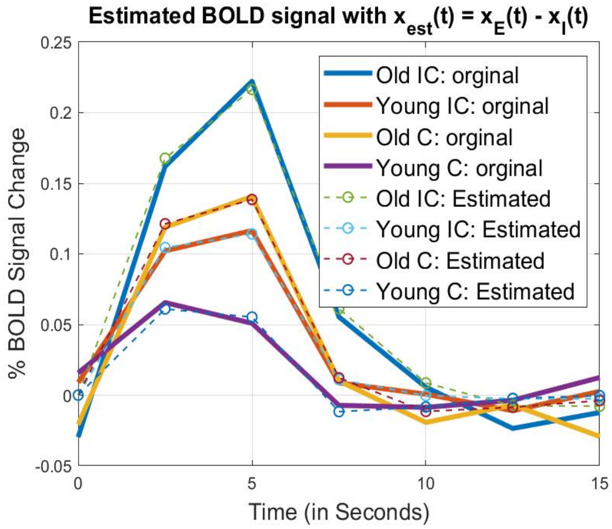

(C) Fixed HRF, IPC-based $x_{est}(t)$

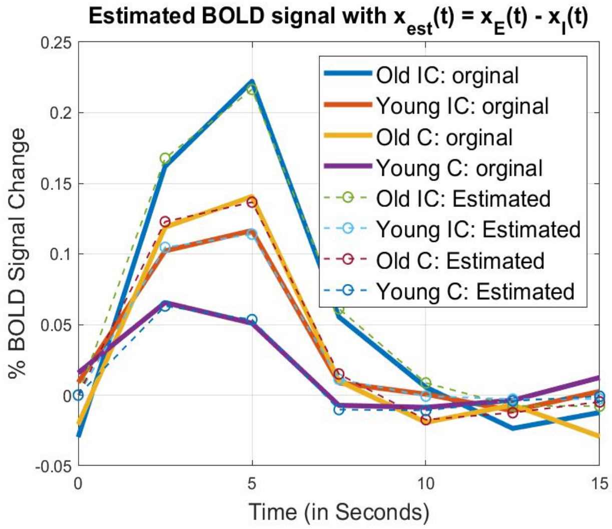

(D) Flexible HRF, IPC-based $x_{est}(t)$

Fig. 5: Results for the right middle frontal gyrus (RMFG) found in the old group.

E. Region name: Left MFG Found in the Old Group

Fixed HRF	m/α	p/α	α/α_C	T_c	T_y	α_1	T_r	BOLD MSE	T_r/T_c
Old IC	3.0536	8.0652	1.6773	0.37861	3.858	4.858	0.995	5.9967e-05	2.6281
Young IC	4.9373	19.737	1.5373	0.25016	2.694	4.858	0.805	4.1844e-05	3.218
Old C	3.8818	13.528	1	0.28695	3.228	4.858	0.789	8.524e-05	2.7496
Young C	7.4832	30.34	1	0.24664	2.34	4.858	0.675	6.1822e-05	2.7368

Flexible HRF	m/α	p/α	α/α_C	T_c	T_y	α_1	T_r	BOLD MSE	T_r/T_c
Old IC	3.0536	8.0652	1.6773	0.37861	3.858	4.858	0.995	5.9967e-05	2.6281
Young IC	5.4101	18.378	1.4486	0.29438	2.694	4.694	0.805	3.8051e-05	2.7346
Old C	3.8818	13.528	1	0.28695	3.228	4.858	0.789	8.524e-05	2.7496
Young C	7.9074	26.623	1	0.29702	2.34	4.69	0.675	5.3629e-05	2.2726

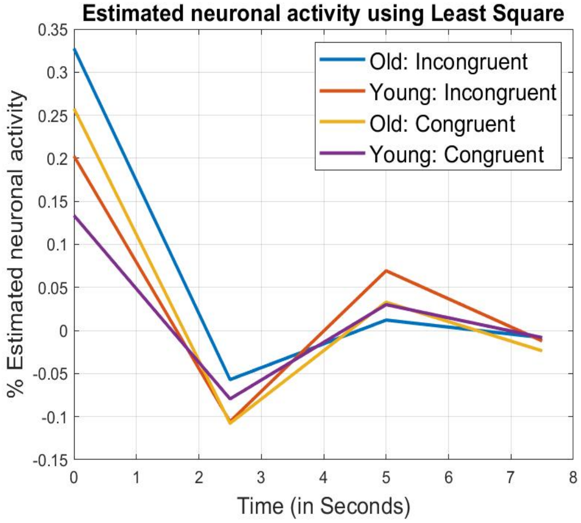

(A) Fixed HRF

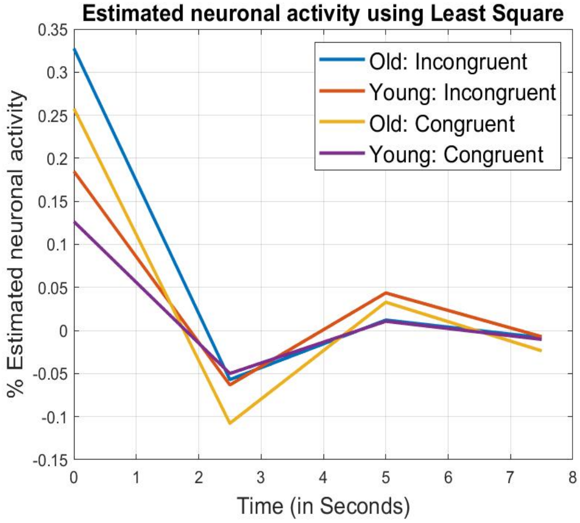

(B) Flexible HRF

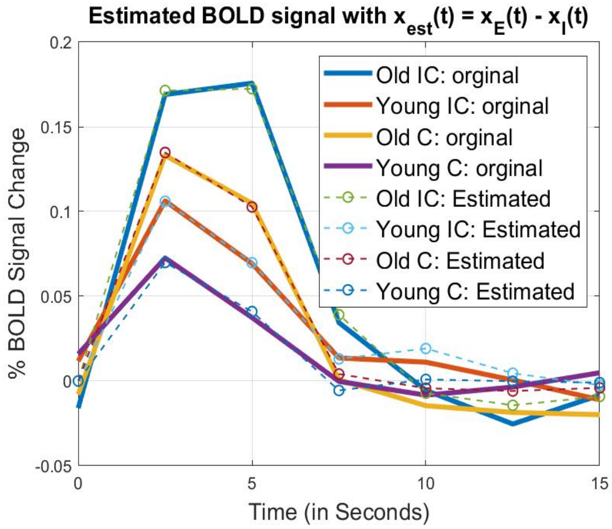

(C) Fixed HRF, IPC-based $x_{est}(t)$

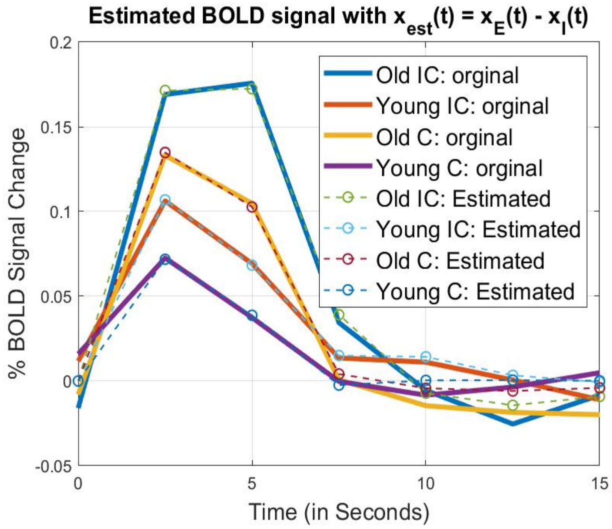

(D) Flexible HRF, IPC-based $x_{est}(t)$

Fig. 6: Results for the left middle frontal gyrus (LMFG) found in the old group.

F. Region name: Left MOG Found in the Old Group

**Fixed HRF**	m/α	p/α	α/α_C	T_c	T_y	α_1	T_r	BOLD MSE	T_r/T_c
Old IC	1.4830	3.8405	1.5377	0.3862	4.438	5.288	0.995	1.1868e-04	2.5767
Young IC	1.7308	4.5928	1.3183	0.3769	4.466	5.288	0.805	4.0405e-05	2.1361
Old C	1.8731	5.9056	1	0.3172	4.174	5.288	0.789	1.8671e-04	2.4876
Young C	2.2790	6.0546	1	0.3764	4.343	5.288	0.675	2.2628e-04	1.7933

**Flexible HRF**	m/α	p/α	α/α_C	T_c	T_y	α_1	T_r	BOLD MSE	T_r/T_c
Old IC	1.4830	3.8405	1.5302	0.3862	4.438	5.288	0.995	1.1868e-04	2.5767
Young IC	1.5956	4.9690	1.3442	0.3211	4.466	5.480	0.805	8.3340e-05	2.5068
Old C	1.8807	5.8768	1	0.3200	4.174	5.278	0.789	1.7952e-04	2.4655
Young C	2.0853	6.6795	1	0.3122	4.343	5.486	0.675	2.1065e-05	2.1622

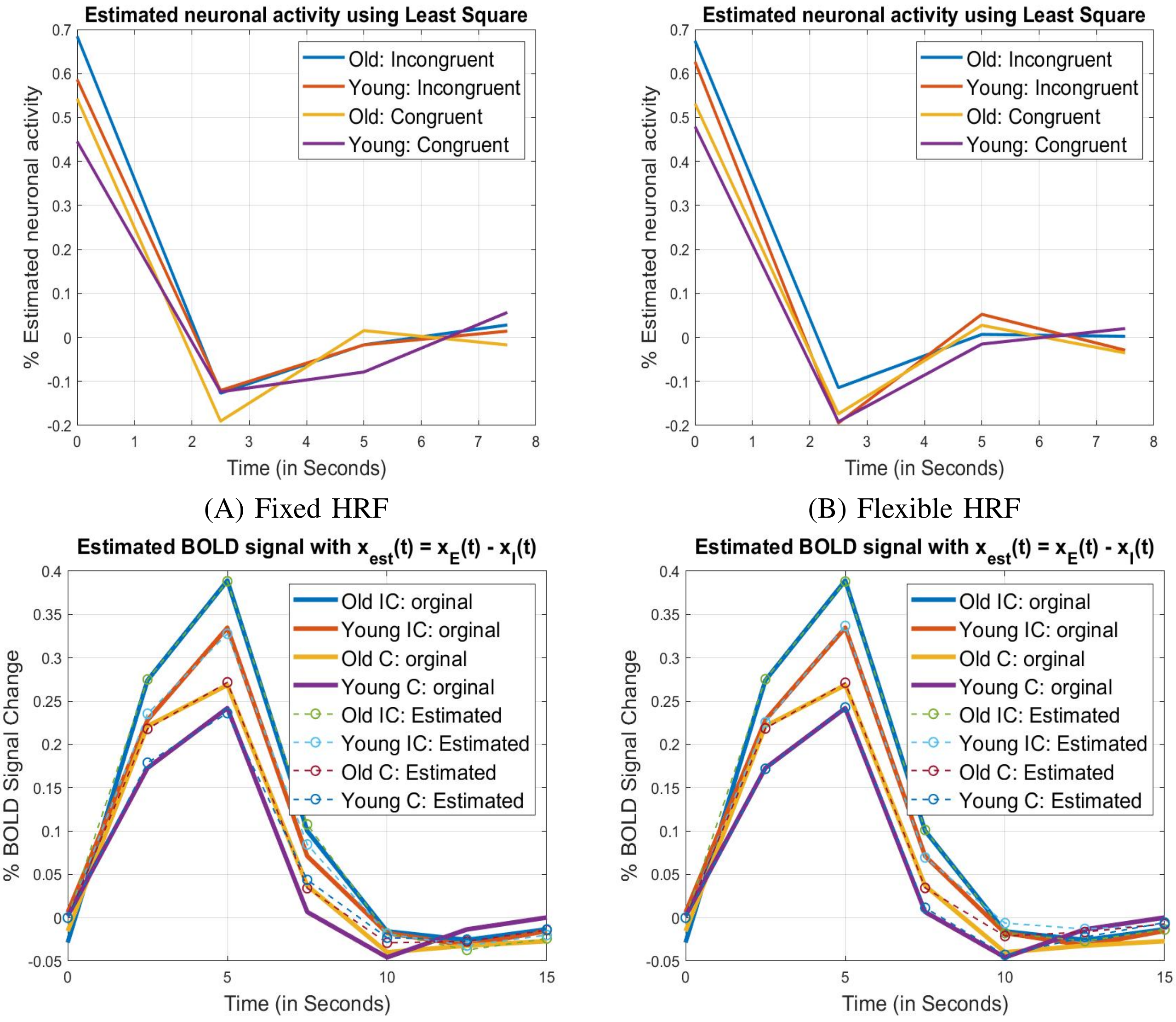

(A) Fixed HRF

(B) Flexible HRF

(C) Fixed HRF, IPC-based $x_{est}(t)$

(D) Flexible HRF, IPC-based $x_{est}(t)$

Fig. 7: Results for the left middle occipital gyrus (LMOG) cluster found in the old group.

G. Region: Left SFG Found only in the Old Group

Flexible HRF	m/α	p/α	α/α_C	T_c	T_y	α_1	T_r	BOLD MSE	T_r/T_c
Old IC	2.0304	5.4727	1.6019	0.37101	3.972	4.972	0.995	2.7437e-04	2.6819
Young IC	3.1654	14.158	1.253	0.22358	2.637	4.972	0.805	2.9161e-04	3.6005
Old C	2.8260	8.7666	1	0.32236	3.586	4.972	0.789	2.1951e-04	2.4476
Young C	3.9554	17.740	1	0.22297	2.281	4.972	0.675	2.3273e-04	3.0273

Flexible HRF	m/α	p/α	α/α_C	T_c	T_y	α_1	T_r	BOLD MSE	T_r/T_c
Old IC	2.0304	5.4727	1.6019	0.37101	3.972	4.972	0.995	2.7437e-04	2.6819
Young IC	3.7237	11.671	1.2623	0.31907	2.637	4.637	0.805	2.2816e-04	2.5230
Old C	2.8260	8.7666	1	0.32236	3.586	4.972	0.789	2.1951e-04	2.4476
Young C	4.4570	14.732	1	0.30254	2.281	4.631	0.675	1.8060e-04	2.2311

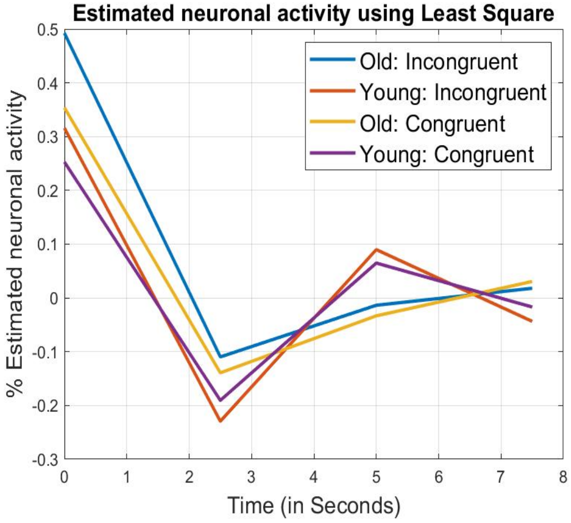

(A) Fixed HRF

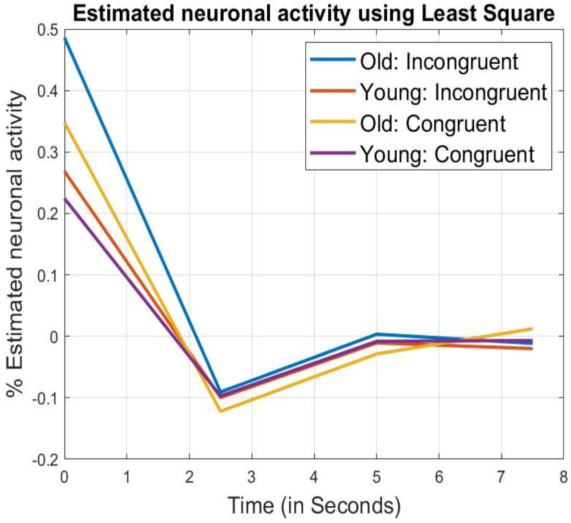

(B) Flexible HRF

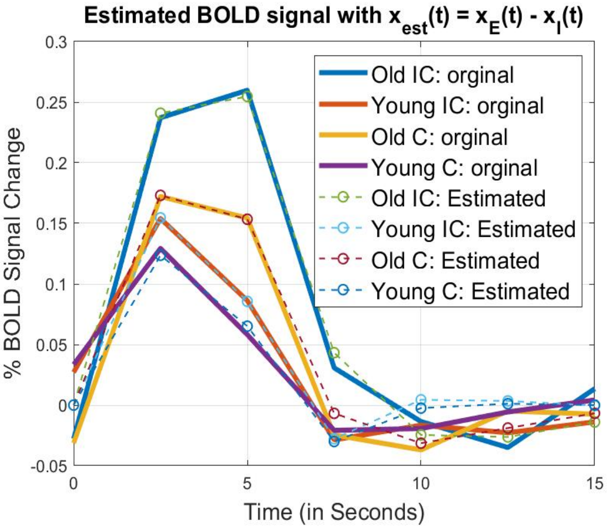

(C) Fixed HRF, IPC-based $x_{est}(t)$

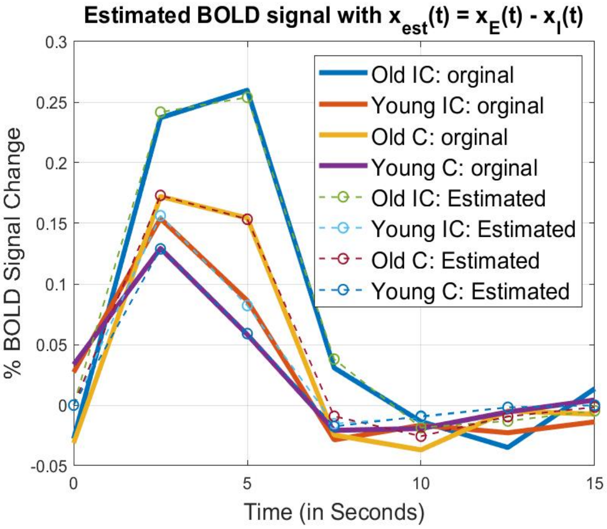

(D) Flexible HRF, IPC-based $x_{est}(t)$

Fig. 8: Results for the left superior frontal gyrus (LSFG) cluster found in the old group.

H. Region: RIFG Found in the Old Group

Flexible HRF	m/α	p/α	α/α_C	T_c	T_y	α_1	T_r	BOLD MSE	T_r/T_c
Old IC	2.1304	6.1605	1.6714	0.34581	4.423	5.423	0.995	9.6023e-05	2.8773
Young IC	3.6553	11.058	1.3455	0.33057	4.37	5.27	0.805	3.6027e-05	2.4352
Old C	3.569	10.297	1	0.34662	4.37	5.22	0.789	7.0028e-05	2.2763
Young C	5.0517	14.878	1	0.33954	4.377	5.467	0.675	3.9768e-05	1.988

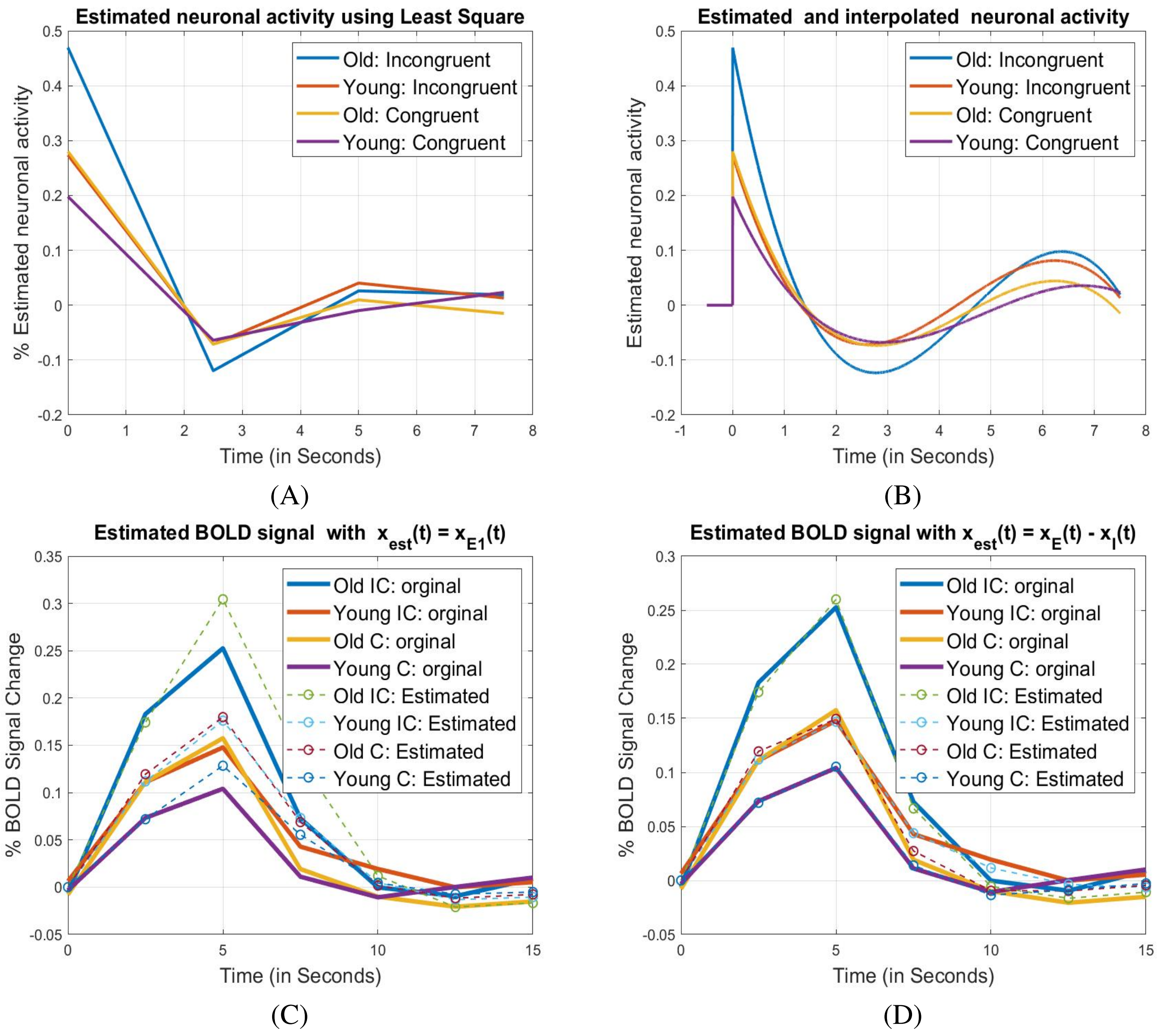

Fig. 9: Results for the right inferior frontal gyrus (RIFG) cluster found in the old group. (A) The estimated neuronal activity using deconvolution with the Least Square method; (B) The interpolated neuronal activity using the cubic spline function in Matlab; (C) Comparison of the original BOLD signal and the estimated BOLD signal obtained using $y_{est}(t) = x_{E1}(t) * h(t)$, where $x_{E1}(t)(t) = \frac{\alpha}{m} e^{-\frac{p}{m}t} u(t)$; (D) Comparison of the original BOLD signal and the estimated BOLD signal obtained using $y_{est}(t) = x_{est}(t) * h(t)$, where $x_{est}(t) = x_E(t) - x_I(t)$.

I. Region: Right IOG Found in the Young Group

**Flexible HRF**	m/α	p/α	α/α_C	T_c	T_y	α_1	T_r	BOLD MSE	T_r/T_c
Old IC	1.3325	3.5178	1.4313	0.37878	4.54	5.39	0.995	9.991e-05	2.6268
Young IC	1.3149	3.8348	1.3937	0.34288	4.591	5.491	0.805	4.3584e-05	2.3478
Old C	1.6644	5.0351	1	0.33055	4.312	5.312	0.789	4.2992e-05	2.3869
Young C	1.7346	5.3446	1	0.32456	4.47	5.49	0.675	5.777e-05	2.0798

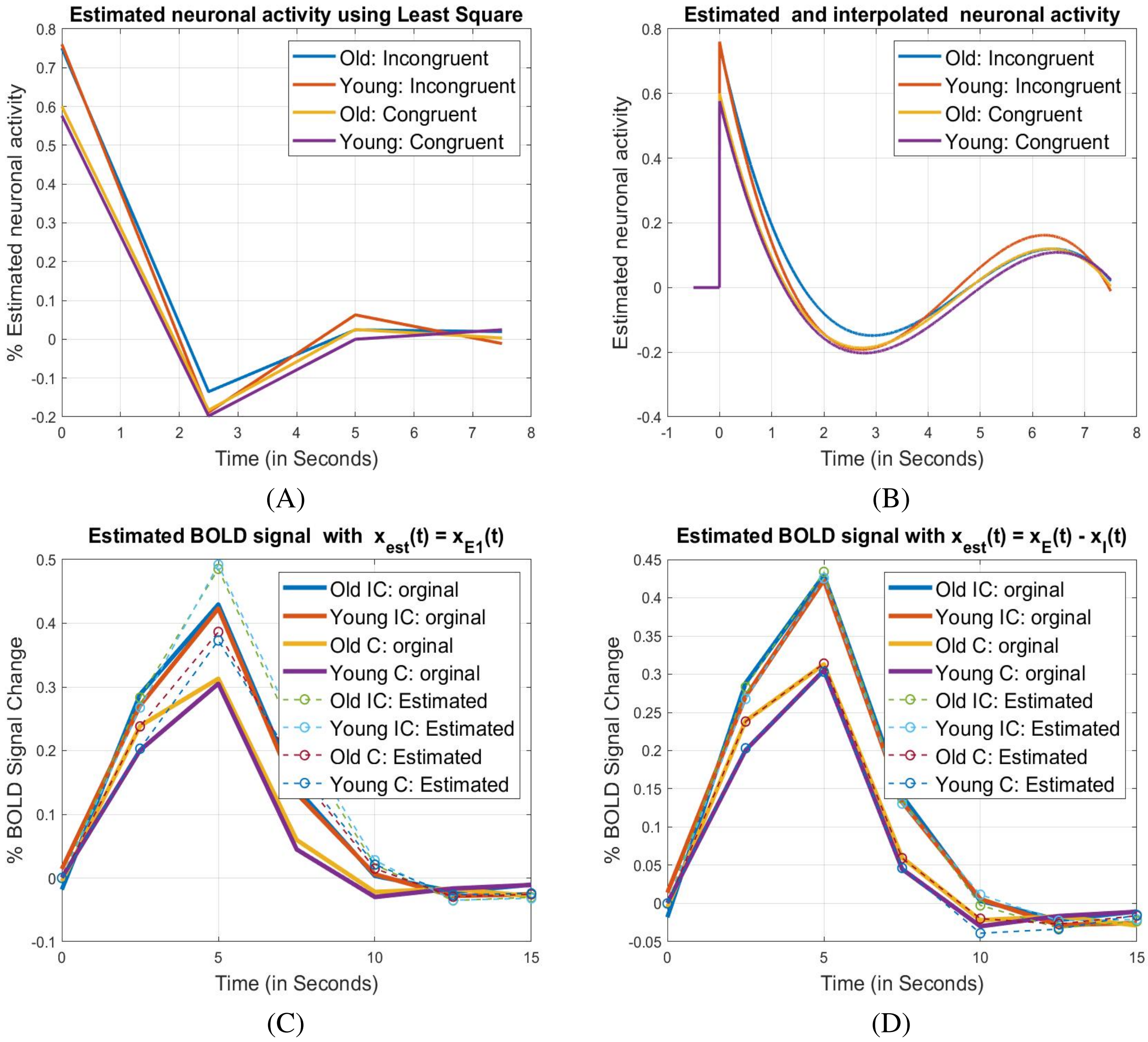

Fig. 10: Results for right inferior occipital gyrus(IOG) cluster found in the young group. (A) The estimated neuronal activity using deconvolution with the Least Square method; (B) The interpolated neuronal activity using the cubic spline function in Matlab; (C) Comparison of the original BOLD signal and the estimated BOLD signal obtained using $y_{est}(t) = x_{E1}(t) * h(t)$, where $x_{E1}(t)(t) = \frac{\alpha}{m} e^{-\frac{p}{m}t} u(t)$; (D) Comparison of the original BOLD signal and the estimated BOLD signal obtained using $y_{est}(t) = x_{est}(t) * h(t)$, where $x_{est}(t) = x_E(t) - x_I(t)$.

J. Region: Right IOG Found in the Old Group

**Flexible HRF**	m/α	p/α	α/α_C	T_c	T_y	α_1	T_r	BOLD MSE	T_r/T_c
Old IC	1.4632	3.9181	1.6875	0.37345	4.797	5.747	0.995	0.00012596	2.6643
Young IC	1.9031	5.3905	1.5206	0.35305	4.757	5.707	0.805	3.2916e-05	2.2801
Old C	2.1851	6.6117	1	0.33049	4.595	5.595	0.789	6.0652e-05	2.3873
Young C	2.7052	8.1967	1	0.33004	4.571	5.651	0.675	0.00012163	2.0452

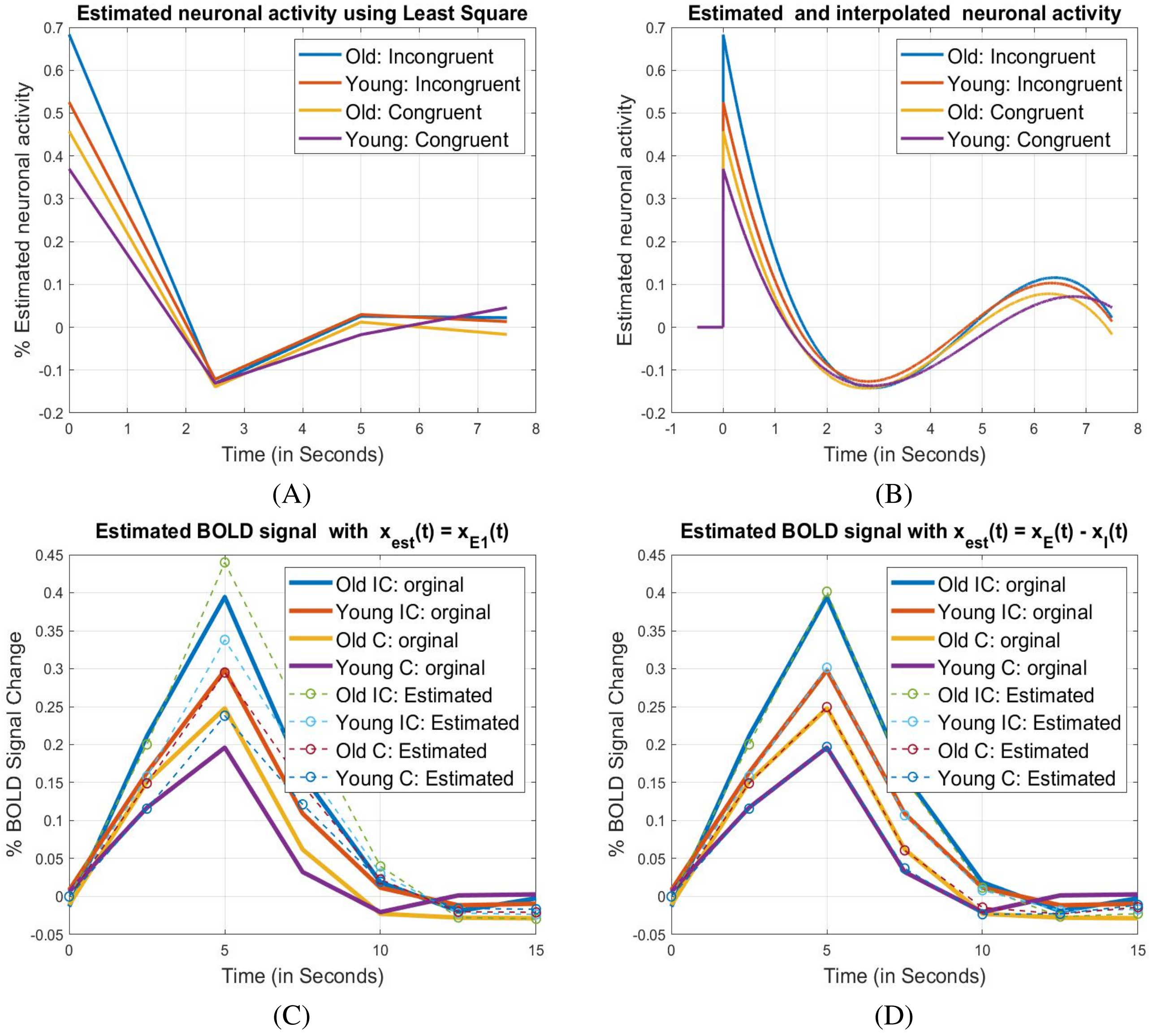

Fig. 11: Results for right inferior occipital gyrus(IOG) cluster found in the old group. (A) The estimated neuronal activity using deconvolution with the Least Square method; (B) The interpolated neuronal activity using the cubic spline function in Matlab; (C) Comparison of the original BOLD signal and the estimated BOLD signal obtained using $y_{est}(t) = x_{E1}(t) * h(t)$, where $x_{E1}(t)(t) = \frac{\alpha}{m} e^{-\frac{p}{m}t} u(t)$; (D) Comparison of the original BOLD signal and the estimated BOLD signal obtained using $y_{est}(t) = x_{est}(t) * h(t)$, where $x_{est}(t) = x_E(t) - x_I(t)$.

K. Behavioral Analysis Results Reporduced from [1]

In [1], Flanker task performance was compared between groups and across conditions using mixed-model ANOVA in which flanker condition (Incongruent versus Congruent) was the repeated-measure factor and age group was the between-group factor. Accuracy (number of correct responses) and response speed (for the correct responses) were analyzed. A Greenhouse-Geisser factor was used to correct for sphericity. Statistical significance was set at $p < 0.05$. The result is reproduced in the table below from [1].

Flanker Task Response Time and Accuracy Results

	**Young Group**	**Old Group**
Response Time (ms)$^{+}$		
Neutral (N)*	671 ± 120	775 ± 98
Congruent (C)	675 ± 119	789 ± 99
Incongruent (IC)	805 ± 188	995 ± 206
Flanker effect (IC-C)	130 ± 92	206 ± 146
Accuracy (% correct)$^{+}$		
Neutral (N)*	97.5 ± 5.2	94.3 ± 9.3
Congruent (C)	97.8 ± 6.1	93.7 ± 10.0
Incongruent (IC)	96.8 ± 6.7	90.0 ± 13.9
Flanker effect (IC-C)	−1.0 ± 1.8	−3.7 ± 7.0

Notes:

*Neutral condition results are included only for informational purposes

$^{+}$Data are presented as means ± standard deviation; response time results include only correct responses.

III. Simulation Results for Individual Subject Pairs

To examine whether the IPC model can distinguish the differences in individuals, we selected two subjects from each group, i.e., one pair from the young group and one pair from the old group. Within each pair, one subject has faster response than the other (as reflected in the response times). For each pair, we applied the IPC model to all the regions that were identified to be active during the flanker test, as well as the averaged BOLD signal across all the regions.

We found that for both the young and old individual pairs, the IPC model was able to show that the faster subject has higher processing capacity and smaller time constant than the slow subject, in most brain regions, (and the average across all regions). It was also observed that compared to the group average, the BOLD signals for individual subjects are much noisier. In some regions (e.g., the right inferior frontal gyrus (IFG) for the young pair), the BOLD signals were considered too noisy for further processing, suggesting that an efficient identification of the IPC model may require noise suppression, through pooling data over regions, trials, or subjects. Simulation results are presented below.

A. III.1 Numerical Results for the Young Individual Pair

1) Averaged BOLD Across All Regions in Each Subject

**Young: Flexible HRF**	m/α	p/α	α/α_C	T_c	T_y	α_1	T_r	BOLD MSE	T_r/T_c
Slow IC	2.0481	4.1248	1.3517	0.49653	4.168	5.118	1.065	5.8157e-04	2.1449
Fast IC	2.2212	6.0929	1.3123	0.36455	4.669	5.960	0.588	5.4610e-04	1.6129
Slow C	2.6719	5.5754	1	0.47923	4.218	5.365	0.903	1.2519e-03	1.8843
Fast C	2.5711	7.9957	1	0.32156	4.561	5.960	0.562	7.3707e-04	1.7477

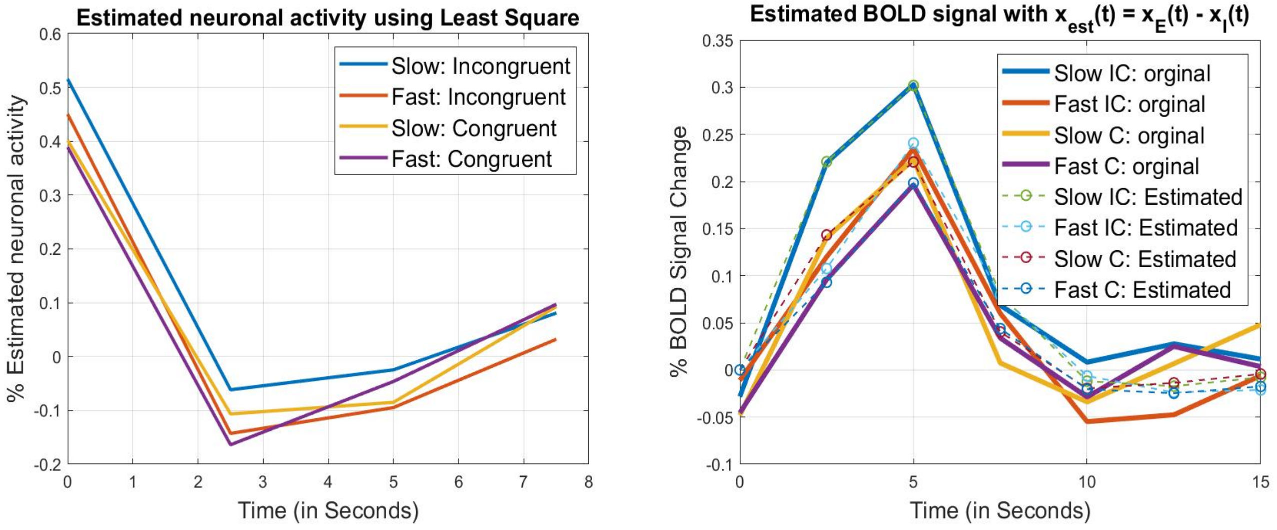

(A) (B)

Fig. 12: Young individuals: results for averaged BOLD across all regions found in the Young subjects. (A) The estimated neuronal activity using deconvolution with the Least Square method; (B) Comparison of the original BOLD signal and the estimated BOLD signal obtained using $y_{est}(t) = x_{est}(t) * h(t)$, where $x_{est}(t) = x_E(t) - x_I(t)$.

2) Left Middle Frontal Gyrus (LMFG)

**Flexible HRF**	m/α	p/α	α/α_C	T_c	T_y	α_1	T_r	BOLD MSE	T_r/T_c
Slow IC	2.0542	5.1963	1.3501	0.39533	3.155	4.305	1.065	1.2835e-03	2.694
Fast IC	2.4928	8.3295	1.2637	0.29927	3.808	5.808	0.588	1.7117e-03	1.9648
Slow C	2.328	7.0155	1	0.33184	3.289	4.639	0.903	1.2253e-03	2.7212
Fast C	2.8625	10.526	1	0.27194	3.993	5.993	0.562	5.5208e-04	2.0666

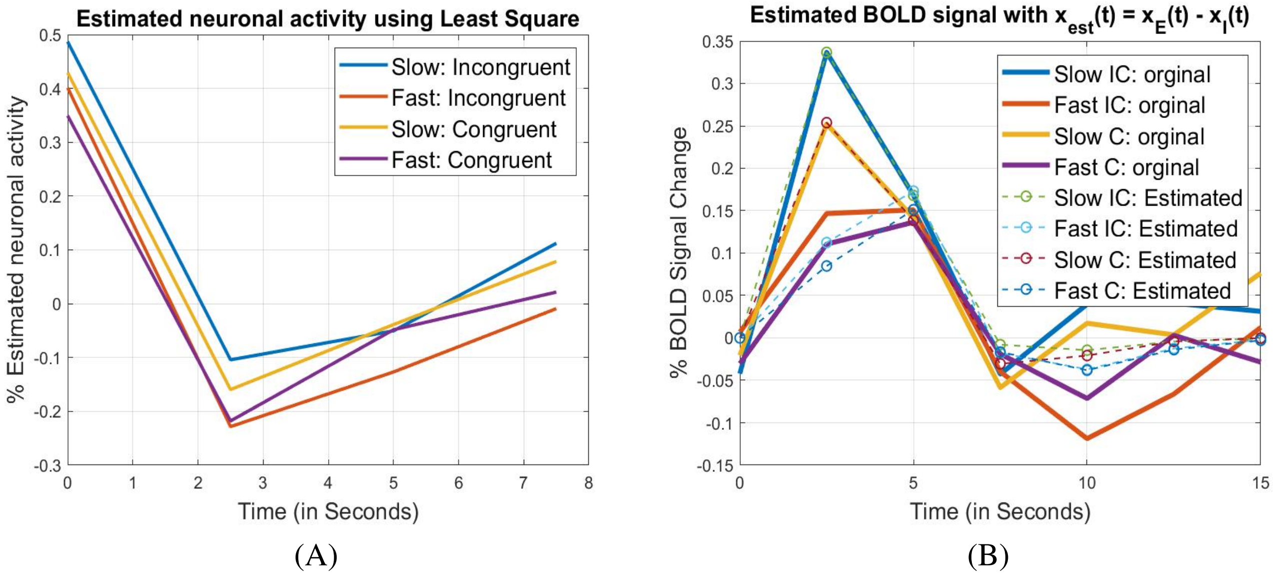

Fig. 13: Young individuals: results for LMFG. (A) The estimated neuronal activity using deconvolution with the Least Square method; (B) Comparison of the original BOLD signal and the estimated BOLD signal obtained using $y_{est}(t) = x_{est}(t) * h(t)$, where $x_{est}(t) = x_E(t) - x_I(t)$.

3) *Right Middle Frontal Gyrus (RMFG)*

**Flexible HRF**	m/α	p/α	α/α_C	T_c	T_y	α_1	T_r	BOLD MSE	T_r/T_c
Slow IC	2.2574	4.7353	1.2787	0.47672	3.378	4.378	1.065	4.7767e-04	2.234
Fast IC	9.9044	27.867	1.3628	0.35541	3.9	5.4	0.588	1.3964e-03	1.6544
Slow C	3.0807	6.0551	1	0.50877	3.535	4.535	0.903	7.4858e-04	1.7749
Fast C	11.704	41.165	1	0.28431	4.932	6.402	0.562	4.9583e-04	1.9767

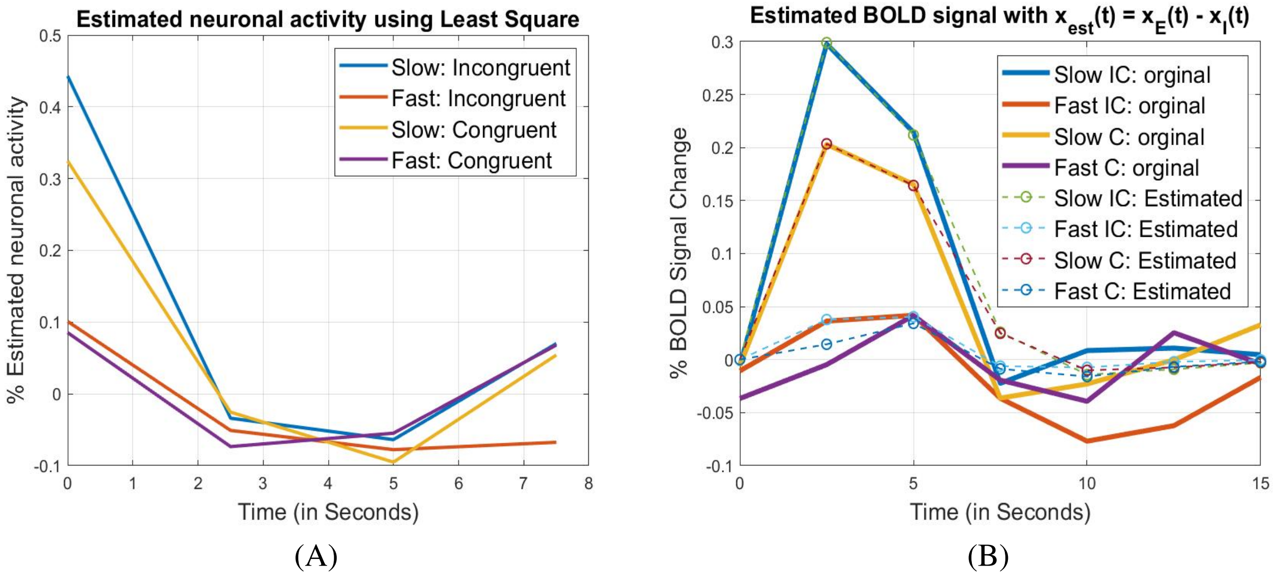

Fig. 14: Young individuals: results for RMFG.

4) Left Lingual Gyrus (LLG)

**Flexible HRF**	m/α	p/α	α/α_C	T_c	T_y	α_1	T_r	BOLD MSE	T_r/T_c
Slow IC	1.3593	3.171	1.2556	0.42869	4.781	5.801	1.065	7.7835e-04	2.4843
Fast IC	1.6303	3.7377	1.2035	0.43617	4.819	5.809	0.588	1.2086e-04	1.3481
Slow C	1.7401	3.9813	1	0.43706	4.558	5.683	0.903	2.7358e-03	2.0661
Fast C	1.7743	4.4983	1	0.39444	4.522	5.652	0.562	5.5799e-04	1.4248

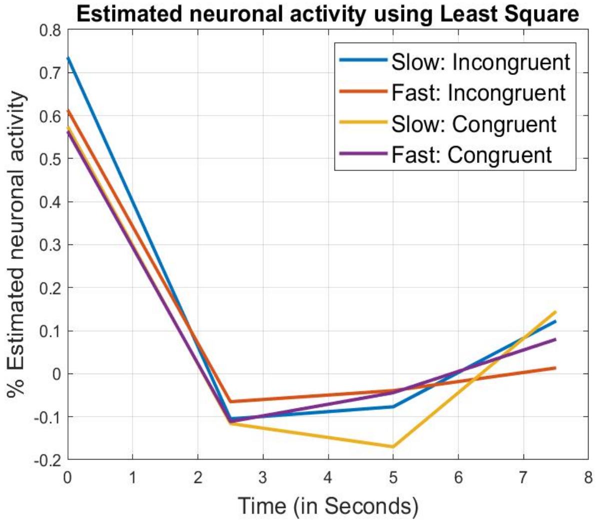

(A)

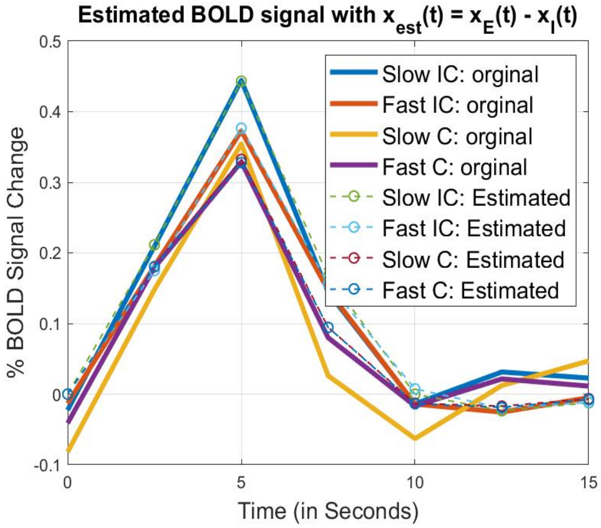

(B)

Fig. 15: Young individuals: results for LLG. (A) The estimated neuronal activity using deconvolution with the Least Square method; (B) Comparison of the original BOLD signal and the estimated BOLD signal obtained using $y_{est}(t) = x_{est}(t) * h(t)$, where $x_{est}(t) = x_E(t) - x_I(t)$.

5) The Right Inferior Frontal Gyrus (IFG)

For the selected Young invidvidual pairs, the BOLD signals in the right IFG are too noisy and irregular for further processing. Please refer to Fig.16.

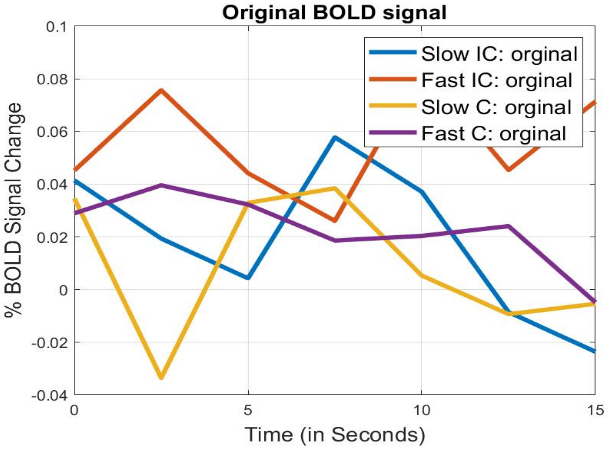

Fig. 16: Young individuals: BOLD signals in Right IFG are too noisy for further processing.

B. Simulation Results for Individual Subjects in the Old Group

1) Averaged BOLD Across All Regions

Old Pair: Flexible HRF	m/α	p/α	α/α_C	T_c	T_y	α_1	T_r	BOLD MSE	T_r/T_c
Slow IC	1.5527	4.1919	1.3286	0.37041	4.937	6.002	1.02	3.5786e-04	2.7537
Fast IC	1.8141	5.4762	1.1806	0.33127	3.857	5.307	0.822	2.1303e-04	2.4814
Slow C	1.9498	5.5694	1	0.35009	4.54	5.64	0.849	1.6986e-04	2.4251
Fast C	2.1097	6.4649	1	0.32632	3.487	4.787	0.71	2.9816e-05	2.1758

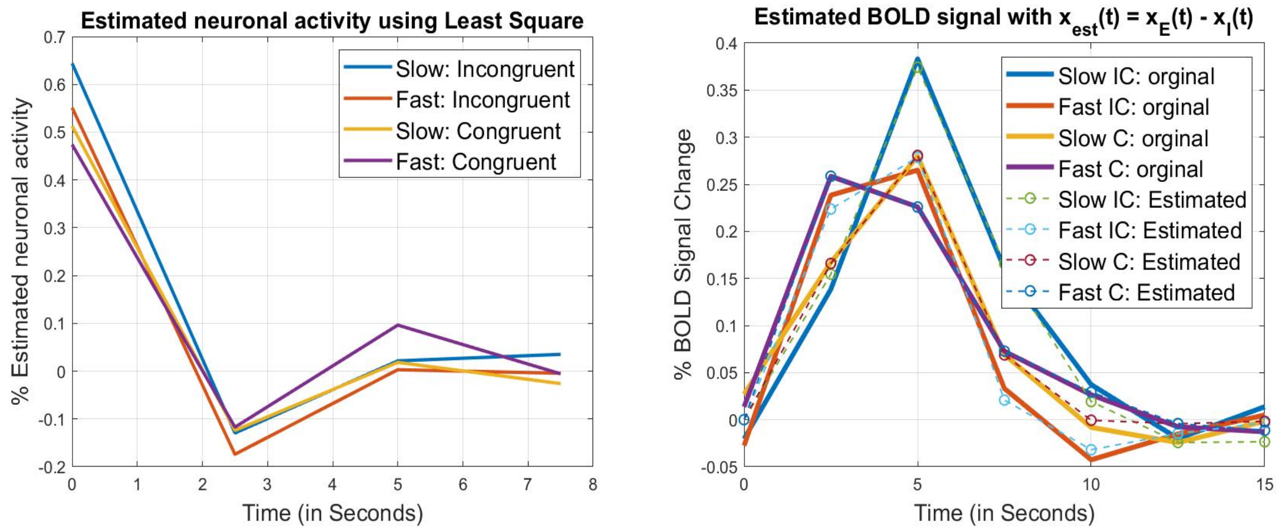

(A) (B)

Fig. 17: Old individuals: results for averaged BOLD across all regions found in the Old subjects. (A) The estimated neuronal activity using deconvolution with the Least Square method; (B) Comparison of the original BOLD signal and the estimated BOLD signal obtained using $y_{est}(t) = x_{est}(t) * h(t)$, where $x_{est}(t) = x_E(t) - x_I(t)$.

2) Left Middle Occipital Gyrus (LMOG)

Flexible HRF	m/α	p/α	α/α_C	T_c	T_y	α_1	T_r	BOLD MSE	T_r/T_c
Slow IC	1.0766	2.9799	1.1849	0.36128	4.455	5.555	1.02	2.9169e-03	2.8233
Fast IC	1.1372	3.2457	1.5518	0.35036	3.726	5.076	0.822	1.1218e-03	2.3462
Slow C	1.1168	3.5309	1	0.31629	4.31	5.81	0.849	1.5359e-03	2.6842
Fast C	1.5021	5.0367	1	0.29823	3.699	5.099	0.71	9.287e04	2.3807

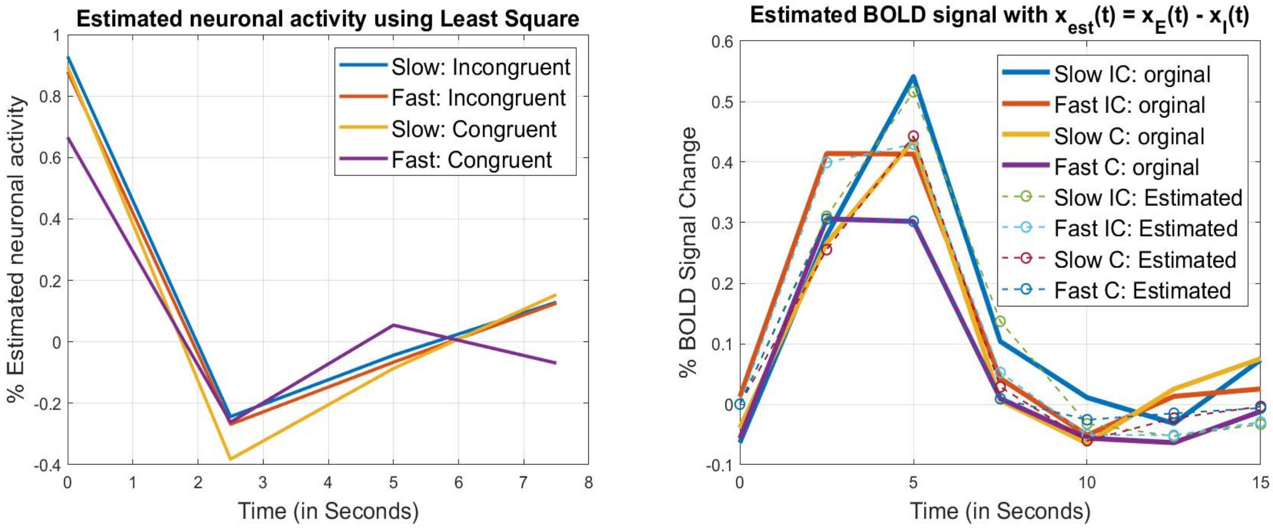

(A) (B)

Fig. 18: Old individuals: results for LMOG. (A) The estimated neuronal activity using deconvolution with the Least Square method; (B) Comparison of the original BOLD signal and the estimated BOLD signal obtained using $y_{est}(t) = x_{est}(t) * h(t)$, where $x_{est}(t) = x_E(t) - x_I(t)$.

3) *Right Inferior Occipital Gyrus (RIOG)*

**Flexible HRF**	m/α	p/α	α/α_C	T_c	T_y	α_1	T_r	BOLD MSE	T_r/T_c
Slow IC	1.3434	3.043	1.3511	0.44146	5.092	6.042	1.02	6.8201e-04	2.3105
Fast IC	1.5117	3.3535	1.6044	0.45079	4.336	5.386	0.822	8.0892e-04	1.8235
Slow C	1.6778	4.1115	1	0.40807	4.872	5.872	0.849	6.5752e-04	2.0805
Fast C	1.8004	5.3804	1	0.33463	4.128	5.208	0.71	7.2047e-05	2.1217

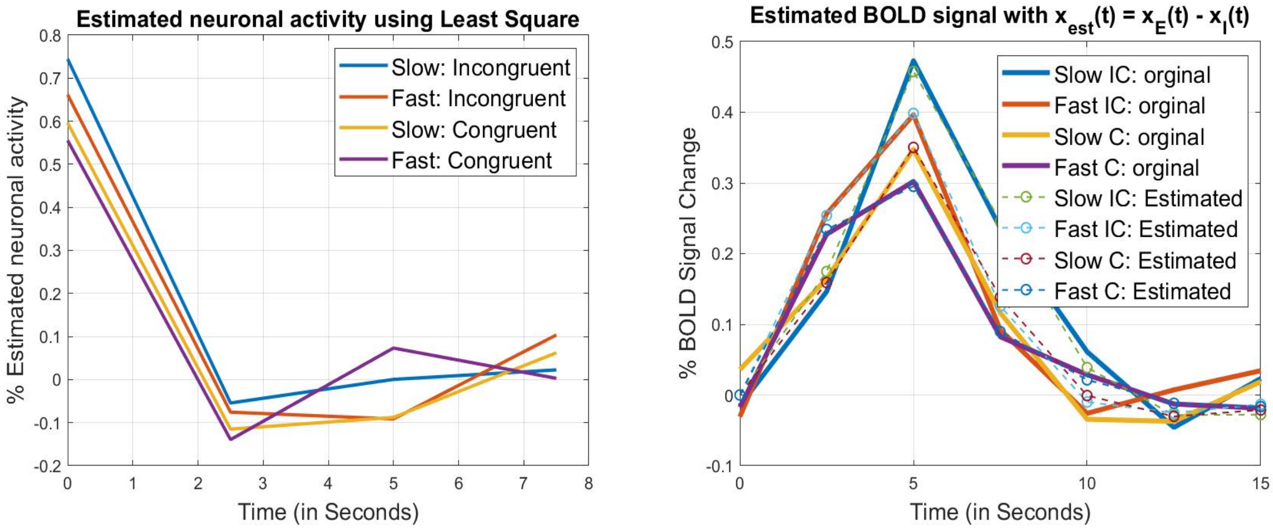

(A) (B)

Fig. 19: Old individuals: results for RIOG. (A) The estimated neuronal activity using deconvolution with the Least Square method; (B) Comparison of the original BOLD signal and the estimated BOLD signal obtained using $y_{est}(t) = x_{est}(t) * h(t)$, where $x_{est}(t) = x_E(t) - x_I(t)$.

4) *Right Inferior Parietal Lobule (IPL)*

**Flexible HRF**	m/α	p/α	α/α_C	T_c	T_y	α_1	T_r	BOLD MSE	T_r/T_c
Slow IC	3.4846	3.5703	1.5052	0.976	6.024	6.874	1.02	0.0042804	1.0451
Fast IC	3.9711	7.4705	1.3221	0.53157	3.918	4.918	0.822	0.0012258	1.5464
Slow C	4.026	5.3741	1	0.74914	5.943	6.874	0.849	0.0026594	1.1333
Fast C	2.6838	9.8764	1	0.27174	3.264	4.868	0.71	0.0012159	2.6128

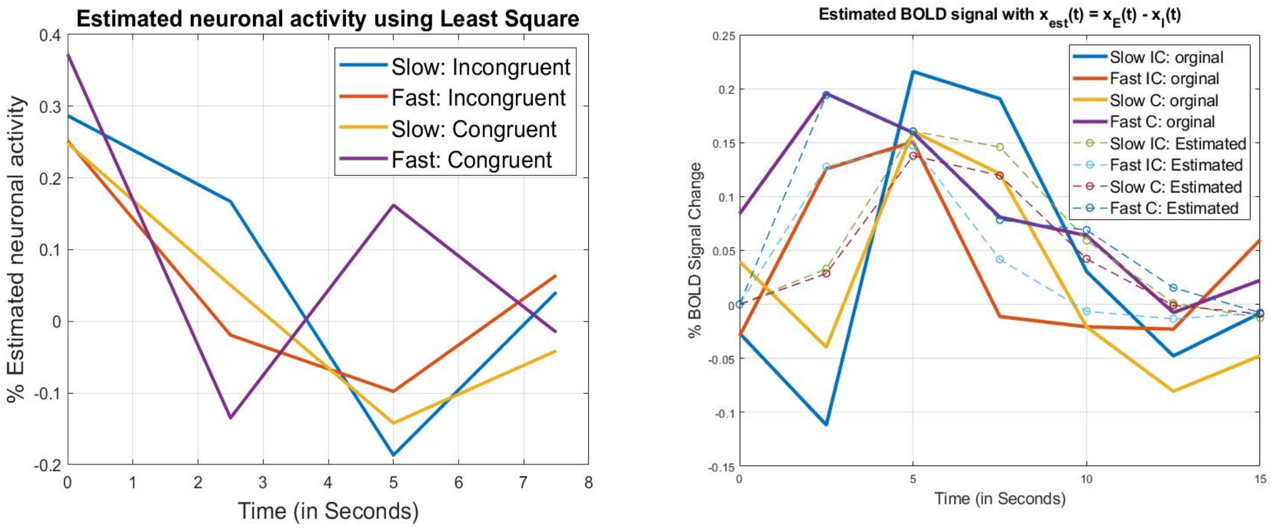

(A) (B)

Fig. 20: Old individuals: results for RIPL. (A) The estimated neuronal activity using deconvolution with the Least Square method; (B) Comparison of the original BOLD signal and the estimated BOLD signal obtained using $y_{est}(t) = x_{est}(t) * h(t)$, where $x_{est}(t) = x_E(t) - x_I(t)$.

REFERENCES

[1] Zhu, D.C., Zacks R.T., Slade J.M., Brain activation during interference resolution in young and older adults: an fMRI study. Neuroimage 50, 810 (2010).

[2] The SPM hemodynamic response function in Matlab, http://web.mit.edu/seven/src/AFNI/matlab/spm_hrf.m

[3] Liao, C. H., Worsley, K. J., Poline, J. B., Aston, J. A., Duncan, G. H., Evans, A. C. Estimating the delay of the fMRI response. NeuroImage, 16(3 Pt 1), 593–606. (2002). https://doi.org/10.1006/nimg.2002.1096

[4] Saad, Z. S., DeYoe, E. A., Ropella, K. M. Estimation of FMRI response delays. NeuroImage, 18(2), 494–504 (2003). https://doi.org/10.1016/s1053-8119(02)00024-1
